# Expression signature and molecular basis of CDH11 in OSCC detected by a combination of multiple methods

**DOI:** 10.1186/s12920-023-01499-7

**Published:** 2023-04-03

**Authors:** Yuxing Wei, Xujie Cheng, Limei Deng, Hao Dong, Huiping Wei, Cheng Xie, Yangjuan Tuo, Guangyu Li, Dahai Yu, Yong Cao

**Affiliations:** 1 Department of Oral and Maxillofacial Surgery, Guangxi Academy of Medical Sciences, The People’s Hospital of Guangxi Zhuang Autonomous Region, Nanning, 530021 China; 2 Department of Stomatology, People’s hospital of Yongning District, Nanning, 530200 China; 3 Department of Stomatology, The First Affiliated Hospital of Guangxi Medical University, Nanning, 530021 China

## Abstract

Oral squamous cell carcinoma (OSCC) is one of the most common malignancy in the oral cancer threatening human health and the survival rate of OSCC has not been effectively improved in recent decades, so more effective biomarkers for the targeted therapy of OSCC are needed. Moreover, the role of CDH11 in OSCC has not been intensively investigated. We here show that the CDH11 protein and mRNA expression levels in the OSCC tissues were all significantly higher than in the non-cancerous tissues using RT-qPCR and western blot. This study also revealed that patients with higher CDH11 levels showed a higher incidence of perineural invasion and lymph node metastasis. By using data available from the Cancer Genome Atlas (TCGA), Gene Expression Omnibus (GEO), and ArrayExpress databases, overexpressed CDH11 in OSCC that associated with patients’history of alcohol, negative Human Papilloma Virus (HPV) status, perineural invasion, infiltration of multiple immune cells, and Single-cell functional states including quiescence and angiogenesis, possessed an excellent discriminatory capability in the OSCC patients. Moreover, the majority of the biological processes or pathways were significantly clustered by co-expressed genes, including extracellular matrix organization, the epithelial to mesenchymal transition, carbon metabolism, and the PI3K-Akt signaling pathway, and the upstream transcriptional regulation mechanism of CDH11 in OSCC was showed on a transcription factor/miRNA-mRNA network with the online tool NetworkAnalyst. Finally, frequent mutation of CDH11 was observed on a mouse OSCC model through whole-genome sequencing. CDH11 might serve as a valuable biomarker in OSCC, as it was identified to be overexpressed in OSCC and related to its clinical progression.

**Supplementary Information:**

The online version contains supplementary material available at 10.1186/s12920-023-01499-7.

## Introduction

Oral cancer is the 11th leading malignancy worldwide. OSCC normally accounts for more than 80% of all oral cancer cases, making it one of the most common malignancies in oral cancer [1]. Data from Global Cancer Statistics in 2018 shows that there are 354,864 new cases of oral cancer worldwide—accounting for 2% of systemic malignancies—with the incidence in developing countries lower than that in developed countries [2]. It can occur anywhere in the mouth, including the tongue, gums, floor of the mouth, palate, and buccal mucosa.

Oral cancer patients may suffer from significant dysfunction in terms of talking and swallowing, alteration in their cosmetic appearance, sensory impairment, and chronic pain, all of which have a negative impact on their quality of life [3]. The overall five-year survival rate for OSCC has been less than 50% in the last three decades—the lowest among all malignancies—despite progress made in the diagnosis and treatment of cancer [4]. Thus, the discovery of effective biomarkers is of great value for targeted OSCC therapy.

CDH11 is a classic type II cadherin that participates in cell adhesion with a complete membrane protein while also playing a role in bone formation and functional stability. CDH11 has been reported to promote the invasion and metastasis of prostate cancer and breast cancer [5, 6]. In other cancers, such as gastric cancer and retinoblastoma, the CDH11 gene is hypermethylated and silenced, functioning as a tumor suppressor [7, 8]. Piao et al. showed that CDH11 was highly expressed in head and neck squamous cell carcinoma tissues. Also, CDH11 gene knockout significantly increased the proliferation and invasion ability of UM-SCC-29 and UM-SCC-47 cells [9]. Nonetheless, the mechanisms underlying CDH11 in OSCC as well as the role of CDH11 in the carcinogenesis and progression of OSCC remain unclear.

In this paper, we detected CDH11 expression in OSCC tissues using RT-qPCR, western blot and immunohistochemistry. The expression levels of CDH11 in OSCC patients contrasted with non-cancer groups was evaluated by integrating multi-center microarrays, a RNA-seq dataset, and in-house RT-qPCR data. The prognostic capacity of CDH11 for OSCC patients was assessed using Kaplan-Meier survival curves. We also explored the potential molecular mechanisms of CDH11 in OSCC and the genomic changes of CDH11 in OSCC by whole-genome sequencing that performed on a mouse OSCC model, hoping to assess if CDH11 can be considered to be a reliable marker of OSCC.

## Materials and methods

### In-house RT-qPCR

Fresh specimens of 20 cases of primary lesions from OSCC patients who underwent combined treatment of the tongue, jaw, and neck roots and as well as matched normal tongue mucosa adjacent to the cancer were collected in the oral and maxillofacial surgery operating room of the People’s Hospital of Guangxi Zhuang Autonomous Region. After the tissue was cut and dried with wet gauze, it was stored in a tissue RNA preservation solution (Vazyme, Nanjing, China) and placed in a -80 ℃ ultra-low temperature refrigerator for RT-qPCR experiments. All surgical samples were diagnosed as moderately or highly differentiated OSCC by specialists in oral pathology. RNA was extracted from the 20 samples for the RT-qPCR experiments. The study protocol was ratifiedby the ethics committee of the People’s Hospital of Guangxi Zhuang Autonomous Region. All patients agreed to the use of their surgically removed specimens for this experiment and signed informed consent forms prior to their operations.

RT-qPCR was performed using the 7500 RT-qPCR system (ABI) following the manual’s instructions. GAPDH served as the housekeeping gene for CDH11. The primer sequences of GAPDH and CDH11 used for the experiments were CTCGCTTCGGCAGCACA (forward sequences for GAPDH), AACGCTTCACGAATTTGCGT (reverse sequences for GAPDH), TGGGCTTCCAGCTGATGCTAT (forward sequences for CDH11), and GTAGCCACCACATAGAGGAAAGG (reverse sequences for CDH11). The formula for 2-△△C was utilized to calculate the expression value of CDH11 in the OSCC and paired normal tongue mucosa tissues.

### In-house western blot assay

Among the above-mentioned 20 cases of primary lesions from the OSCC patients and the paired normal tissues, nine samples—including three OSCC samples from patients with lymph node metastasis, three OSCC samples from patients without lymph node metastasis, and three cases of normal tissues were chosen for western blot analysis. Heretofore, studies have described the exhaustive procedure involved in carrying out a western blot assay. First, the total protein was collected from tissue samples via lysis in radio immunoprecipitation assay (RIPA) buffer, ultrasonic crushing, centrifugation, and separation of the supernatant. Equal amounts of these protein extracts were added to loading holes in 12% SDS-PAGE separation gel. Electrophoresis was conducted under a constant voltage of 80 V until the bromophenol blue ran out of the concentrated gel layer; then, the voltage was adjusted to 120 V. When the bromophenol blue migrated to approximately 1 cm from the lower edge of the separation gel, we turned off the power supply and stopped the electrophoresis. Under the condition of a constant ice bath, the protein mixture was transferred to a PVDF membrane via wet transfer. After the film transfer, the film was sealed with a room-temperature shaking table for 1 h and incubated with primary antibodies against CDH11 (A6-C12, Huaan Biotechnology) as well as horseradish peroxidase (HRP)-conjugated secondary antibodies (7074 S, Cell Signaling Technology). Enhanced chemiluminescence was used for development, and, finally, the gray values of the target strip were calculated via Image J software.

### In-house IHC experiment

A total of 83 OSCC patients treated by oral and maxillofacial surgery at the People’s Hospital of Guangxi Zhuang Autonomous Region were enrolled in the IHC experiments. The inclusion criteria for eligible samples were as follows: (1) The tumors of the OSCC patients occurred in the lip, tongue, palate, floor of the mouth, gingiva, or buccal mucosa; (2) all OSCC cases were first onset, and the patients did not receive radiotherapy and chemotherapy prior to operation; (3) the postoperative pathological diagnosis of all cases was “squamous cell carcinoma”; and (4) the OSCC patients had no history of systemic immune diseases except for oral lesions. This study was ratified by the ethics committee of the People’s Hospital of Guangxi Zhuang Autonomous Region. Paraffin blocks of all included patients’ specimens were found in the specimen bank of the pathology department, and the clinical data of these patients (i.e., age, gender, T-stage, lymph node metastasis and differentiation) were collected from the electronic medical record system. The IHC experiments were carried out in accordance with the aforementioned protocol. The tissue sections were incubated with a rabbit anti-CDH11 polyclonal antibody (CSB-PA005036ESR1HU; 1:100, Cusabio) and a conjugated secondary antibody. The protein expression of CDH11 was observed under a microscope. The staining intensity of the CDH11 protein was scored as 0, 1, 2, or 3—corresponding to no, weak, moderate, and strong expression. No expression (0) or low expression (1) was considered negative expression, and high expression (2–3) was considered positive expression. All IHC scores were assigned by two senior oral pathology experts through consultation.

### Curation of expression data from other microarrays and RNA-seq datasets

We downloaded clinical data and fragments per kilobase per million (FPKM) gene expression matrix for OSCC tissues and non-cancer oral tissues from the TCGA-head and neck squamous cell carcinoma (HNSCC) project in TCGA database. The RNA-seq dataset from the TCGA database was transformed to the data format of transcripts per kilobase million and normalized with the formula of log2 (transcripts per kilobase million value + 0.001). Moreover, microarrays from other databases, such as GEO and ArrayExpress, were also included for differential expression analysis in the current work if they contained a gene expression matrix of no less than three human OSCC and three non-cancer human oral samples (published before June 11, 2021).

### Compilation of CDH11 expression in OSCC data via in-house RT-qPCR, IHC, external RNA-seq dataset, and microarrays

The gene expression matrices in all the included microarrays were corrected using the normalizeBetweenArrays function of limma package and scaled to the log2(x + 0.001) expression matrix when the numerical value of the expression abundance was large. Then, the microarrays were aggregated using the GPL platform, and batch effects were removed through the limma package loaded by R software v.3.6.1. The MRNA or protein expression data and diagnostic CDH11 data in the OSCC and non-cancer oral samples from the in-house RT-qPCR and IHC experiments were merged with that of other public microarray and RNA-seq datasets. A standard mean deviation (SMD) plot for the comprehensive evaluation of the differential expression of CDH11 in the OSCC versus non-cancer oral tissues and the matched summarized receiver’s operating characteristics (SROC) curves was made according to the extracted expression and diagnostic data of CDH11 in the OSCC and non-cancer oral samples [10].

### Overall survival analysis for CDH11 in OSCC

Datasets with OSCC patient survival information and corresponding expression values of CDH11 from the TCGA, GEO, ArrayExpress and other databases were included for prognostic analysis. The overall survival difference between the OSCC patients with high or low CDH11 expression (the median expression value of CDH11 was the threshold value) was analyzed via Kaplan-Meier survival curves with a log-rank test in GraphPad Prism v.8.0.1. The hazard ratio (HR) values with a 95% confidence interval (CI) yielded from the log-rank test for the Kaplan-Meier survival curves were sorted out and summarized by meta package in R software. Significant prognostic value was indicated by p < 0.05.

### Statistical analysis

Differential expression and clinico-pathological significance were analyzed using SPSS 19.0 software. The expression data of CDH11 from the in-house RT-qPCR experiments and other public RNA-seq datasets or microarrays was shown as the mean (M) ± the standard deviation (SD). The RT-qPCR results were first tested for homogeneity of variance. If the variance was homogeneous, a paired sample t-test was used for pairwise comparison. If the variance was uneven, a Wilcoxon paired-sample t-test was used for pairwise comparison. The chi-square test was employed for analyzing the correlation between the protein expression of CDH11 and the clinico-pathological features of the OSCC patients from the in-house cohort. When the CDH11 expression data of the OSCC patients of a certain clinical variable group from the TCGA database conformed to a normal distribution, an independent Student’s t test or analysis of variance (ANOVA) were selected for analyzing the relationship between the CDH11 expression and the clinical features of the OSCC patients in the TCGA database; otherwise, a Mann–Whitney or Kruskal-Wallis test was adopted. Statistical significance was indicated as p < 0.05. All statistical tests were bilateral.

### Up-stream transcriptional regulation network of CDH11 in OSCC

Up-stream miRNAs and transcriptional factors that might potentially regulate the transcription or expression of CDH11 in OSCC were predicted with the online tool NetworkAnalyst (http://www.networkanalyst.ca). A co-regulation network featuring predicted miRNAs, transcription factors, and CDH11 was subsequently built.

### Relationship between CDH11 expression and the proportions of different immune cells in OSCC

We calculated the infiltration levels of the 22 immune cells, including memory B cells, naïve B cells, CD8-positive T cells, plasma cells, and naïve CD4-positive T cells, in the OSCC samples from the TCGA database using the algorithm CIBERSORT in R software v.4.0.1. The association between the CDH11 expression and the percentage of diverse immune cells in OSCC was explored by applying an independent Student’s t test with the purpose of comparing the proportions of the immune cells in the OSCC patient groups divided by the median normalized expression value of CDH11. Significant differential distributions of the immune cells (p < 0.05) were visualized with GraphPad Prism v.8.0.1.

### Functional enrichment of genes co-expressed with CDH11 in OSCC

For all external RNA-seq datasets and microarrays that contained a gene expression matrix of at least three OSCC samples and three non-cancer oral samples, differential expression analysis was performed after processing the raw data with the methods stated in Sect. 2.4 and 2.5. Specifically, limma package and the voom algorithm were utilized for identifying differentially expressed genes (DEGs) in the expression matrix from the microarrays and the count matrix from the RNA-seq data, respectively (|log2FC|&adj.p < 0.05). SMD values (95% CI) were then estimated for all preliminarily screened DEGs based on the normalized expression matrix of all included microarrays and the TPM expression matrix of the RNA-seq dataset. The final DEGs of OSCC met the conditions of significant up-regulations in OSCC samples with positive SMD values (lower limit of 95% CI > 0) or significant down-regulations of OSCC samples with negative SMD values (upper limit of 95%CI < 0). Correlation analysis was also conducted on the normalized expression matrix of all included microarrays and the TPM expression matrix of the RNA-seq dataset for selecting genes significantly correlated with CDH11 expression (adj. p < 0.05). The intersection of the up-regulated final DEGs and genes significantly correlated with CDH11 in more than six datasets or the intersection of the down-regulated final DEGs and genes significantly correlated with CDH11 in more than four datasets were regarded as co-expressed genes of CDH11 in OSCC. The activities of all genes positively or negatively co-expressed with CDH11 in biological processes, cellular components, molecular functions, and KEGG pathways [11–13] were further investigated with ClusterProfiler package in R software v.4.0.1. Significantly enriched terms were indicated by p value < 0.05.

### Animal experiments

#### Mouse oral squamous cell carcinoma model

One-month-old healthy male Balb/c mice weighing about 20 g were selected and supplied by the Animal Experiment Center of Guangxi Medical University. Specifically, 70 BALB/c mice were randomly divided into an administration group and control group. The administration group (n = 60) had 200 mg/L 4-nitroquinoline-1-oxide (4-NQO) added to their drinking water to induce OSCC; this was then changed to normal drinking water after 20 weeks of administration. The control group (n = 10) was given normal drinking water. All experiments in this study were performed in accordance with an ethical permit ratified by The Animal Care & Welfare Commitee of Guangxi Medical University.

#### Sample collection and DNA isolation

All the mice were anesthetized via intraperitoneal injection of 10% chloral hydrate solution (0.003ml/g) and were euthanized using CO_2_ followed by cervical dislocation. The administration group was sacrificed every two weeks from the 21st to the 36th week, while all the mice in the normal group were sacrificed at the 40th week. The 36 Sequencing samples were obtained, including nine normal muscle tissue samples, nine tongue primary tumor tissue samples, six lymph node disseminated tumor cell samples, three lymph node metastatic cancer cell samples, and nine bone marrow disseminated tumor cell samples. According to the pathological stage, the 36 samples were divided into the moderate to severe dysplasia group, the oral squamous cell carcinoma group without lymph node metastasis, and the oral squamous cell carcinoma group with lymph node metastasis. After single-cell suspension of the lymph nodes and bone marrow were prepared, the immune cells in the lymph nodes and bone marrow tissues were dislodged via CD45 magnetic activated cell sorting (MACS), and the tumor cells were enriched by CD326 magnetic beads. DNA extraction and genomic amplification were performed on the disseminated tumor cells (DTCs) using the REPLI-g Single Cell Kit (QIAGEN, Germany). The DNA amplification products of the DTCs were purified using Agencourt AMPure XP magnetic beads (Beckman Genomics, USA). The DNA of the mouse tongue primary focus and normal muscle tissue was extracted using the DNA Mini Kit (QIAGEN, Germany). Finally, the DNA concentration was detected using the Qubit 2.0 Fluorometer (Life Technologies, CA, USA), and the DNA purity was detected using a NanoPhotometer Spectrophotometer (IMPLEN, CA, USA).

#### Analysis of DNA sequences

We took a 1-ug gDNA template to build a sequencing library based on the TruSeq DNA Sample Preparation Guide (Illumina, 15,026,486 Rev. C) method and process. After the library was constructed, we used the Qubit 2.0 Fluorometer (Life Technologies, CA, USA) for preliminary quantification, diluted the library to 1 ng/ul, and then used the Agilent 2100 Bioanalyzer (Agilent Company, Germany) to detect the insert size of the library. After the insert size met the expectations, Q-PCR was performed to accurately quantify the effective concentration of the library (effective concentration of the library > 10 nM) to ensure the quality of the library using the Bio-RAD CFX 96 fluorescent quantitative PCR instrument (Eppendorf, Germany) and Bio-RAD KIT iQ SYBR GRN (Agilent, Germany). The qualified library was run using a paired-end sequencing program (PE150) on the HiSeq X Ten sequencing platform to obtain 150-bp sequence reads. Whole-genome sequencing database construction and computer were completed by Annoyoda (Beijing, China).

### Single-cell functional analysis

The Cancer Single-Cell State Atlas (CancerSEA)[14], a database with the aim of describing the 14 functional states of 41,900 cancer cells from 25 tumor types at the single-cell level, was utilized to achieve a better understanding of the role CDH11 might play in single head and neck cancer (HSNCC) cells.

## Results

### In-house RT-qPCR

**Fig. 1:**
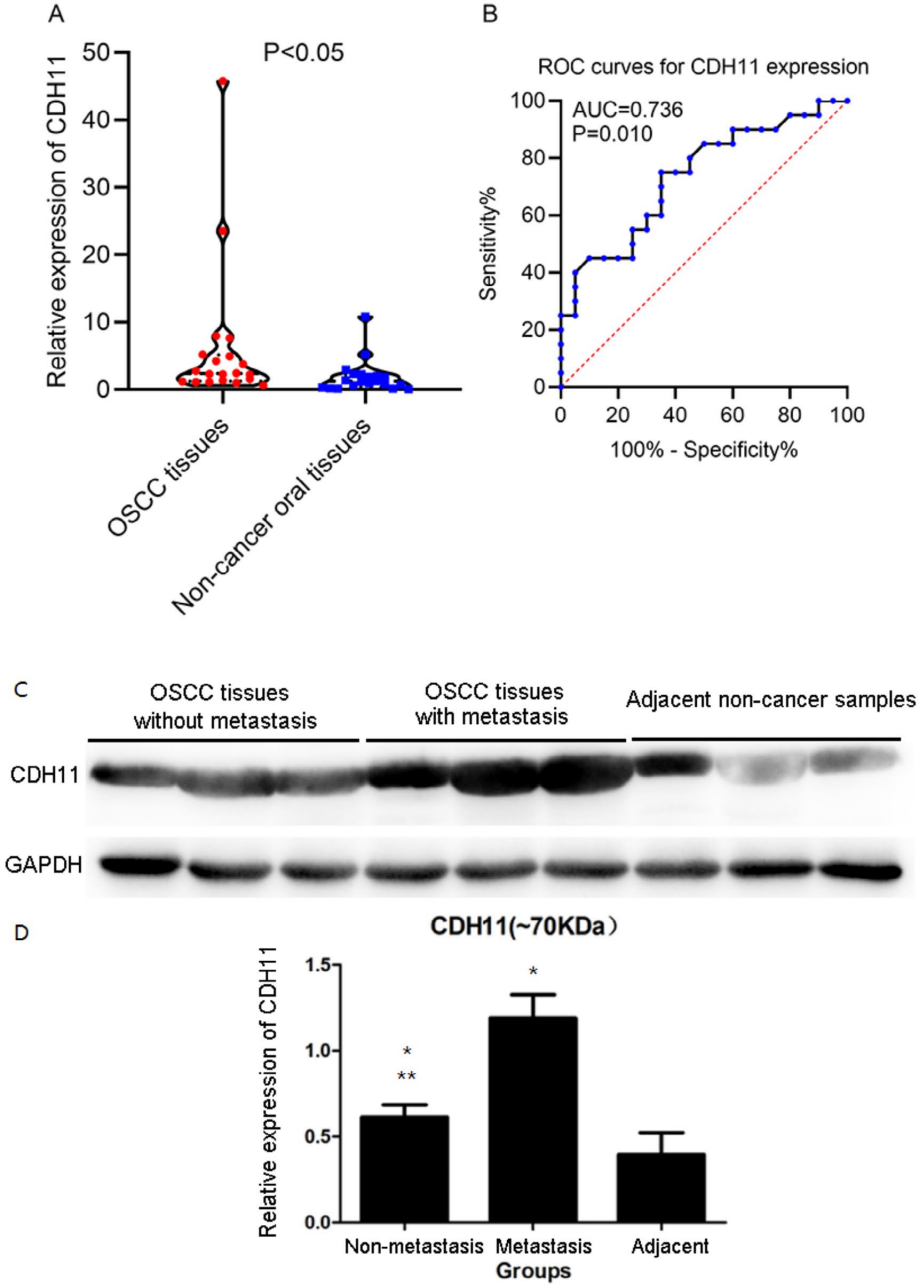
The expression of CDH11 mRNA and protein in oral squamous cell carcinoma based on RT-qPCR and western blot. (A) Scatter plot of CDH11 mRNA expression level of 20 cases from oral squamous cell carcinoma tissues and paired normal tissues. (B) ROC curve; AUC, area under the curve. (C) Western blot results of proteins from different tissues. (D) Relative gray values of western blot in different tissues; * compared with paracancer; ** compared with metastatic group

The expression value from the RT-qPCR showed significantly higher CDH11 expression in the OSCC tissues when compared with the non-cancer oral tissues (Fig. 1) (p = 0.019).

### In-house Western bot assay

The color of the Western blot band of the internal reference protein, GAPDH, was uniform, indicating basically the same amounts of the sample protein (Fig. 1). The results of the gray values from the western blot assay indicate that the expression of CDH11 protein in the OSCC samples was remarkably higher than that in the adjacent non-cancer oral tissues (Fig. 1, p < 0.05), which was consistent with RT-qPCR results. Full-length blots/gels are presented in Figure S1 and Figure S2. Particularly, the CDH11 protein exhibited notably higher expression in the metastatic OSCC tissues than in the non-metastatic OSCC tissues (Fig. 1, p < 0.05).

### In-house IHC experiment

**Fig. 2:**
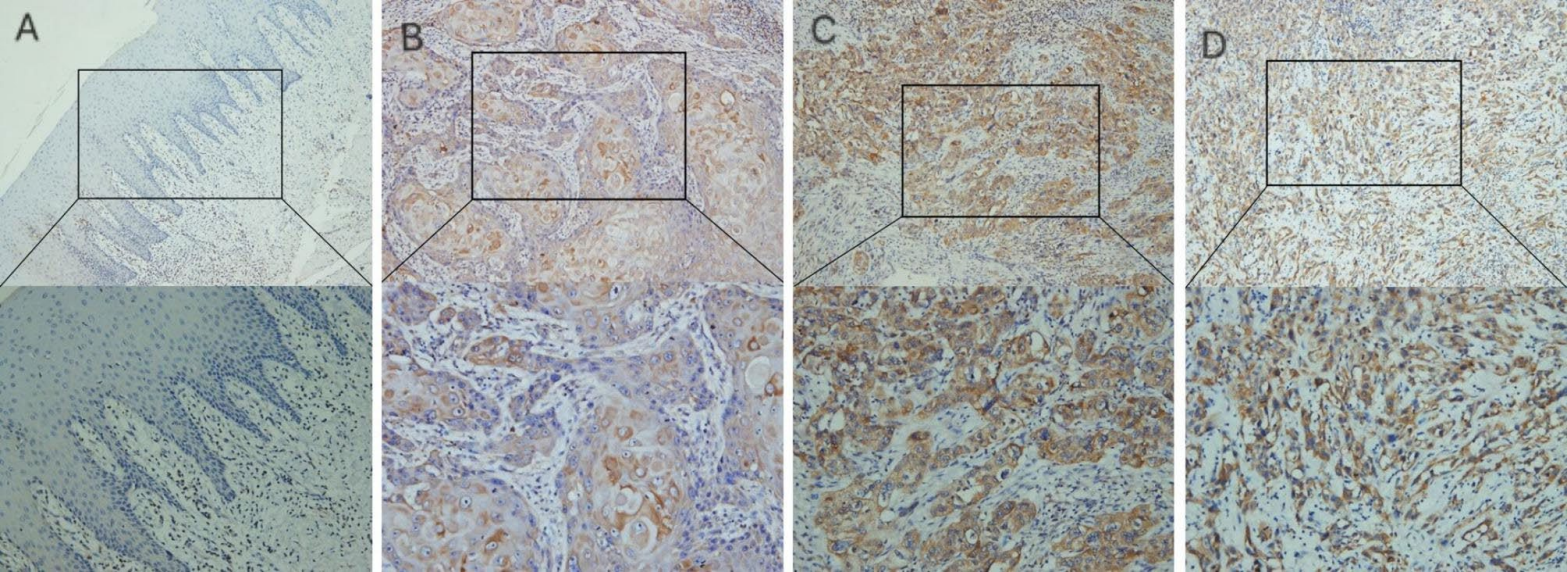
Protein expression of CDH11 in oral squamous cell carcinoma from different tissues based on immunohistochemistry. (A) Immunohistochemical staining of CDH11 protein in normal mucosa (×100) and (×200). (B) Immunohistochemical staining of CDH11 protein in well-differentiated oral squamous cell carcinoma (×100) and (×200). (C) Immunohistochemical staining of CDH11 protein in moderately differentiated oral squamous cell carcinoma (×100) and (×200). (D) Immunohistochemical staining of CDH11 protein in poorly differentiated oral squamous cell carcinoma (×100) and (×200). Staining scores of case (A) was 0. The staining scores of case (B) was 1. The staining scores of case (C) and case (D) were all 2

While the CDH11 expression was negative in the normal oral mucosa tissues, the positive expression rate of CDH11 in the OSCC samples was 51.81% (43/83). Typical IHC staining images of CDH11 in the OSCC and normal oral mucosa tissues are shown in Fig. 2. The positive expression rates of the CDH11 protein in the OSCC patients with lymph node metastasis were 62.16% (N1) and 60.87% (N2) (23/37; 14/23) respectively, which were obviously higher than those in the OSCC patients without lymph node metastasis (N0) (26.09%; 6/23) (p < 0.05) (Table 1). The CDH11 protein expression was also significantly correlated with the perineural infiltration state in the OSCC patients. The positive expression rate of CDH11 in the OSCC patients with perineural infiltration (PNI+) was notably higher (68.97%, 20/29) when compared with the OSCC patients without perineural infiltration (PNI-) (42.60%, 23/54) (p < 0.05) (Table 1). No significant relationships could be found between CDH11 expression and other clinical variables of the OSCC patients.

**Table 1:** The relationships between CDH11 expression and the clinical variables of OSCC

Clinical variables			CDH11				
			Positive	Negative	P value		
Gender		Male	27 16	23 17	0.623		
		Female					
Age		≤ 60	9 34	13 27	0.233		
		>60					
Smoking		Yes No	23 20	22 18	0.890		
Alcohol history		Yes No	28 15	24 16	0.630		
Lymph node metastasis		N0	6 23 14	17 14 9	Vs N1: 0.007^*^ Vs N2: 0.920 Vs N0: 0.017^*^		
		N1					
		N2					
T stage	T1-2		13 30	20 20	0.066		
	T3-4						
Tumor Differentiation	High		32	26	Vs Medium: 0.168		
	Medium		4	8	Vs Low: 0.302		
	Low		7	6	Vs High: 0.931		
PNI		+	20 23	9 31	0.022^*^		
		-					
KI67		+ -	16 27	14 26	0.834		

Note: PNI: perineural infiltration; *: P < 0.05

### The significant aberrant expression of CDH11 between OSCC and non-cancer oral samples

**Fig. 3:**
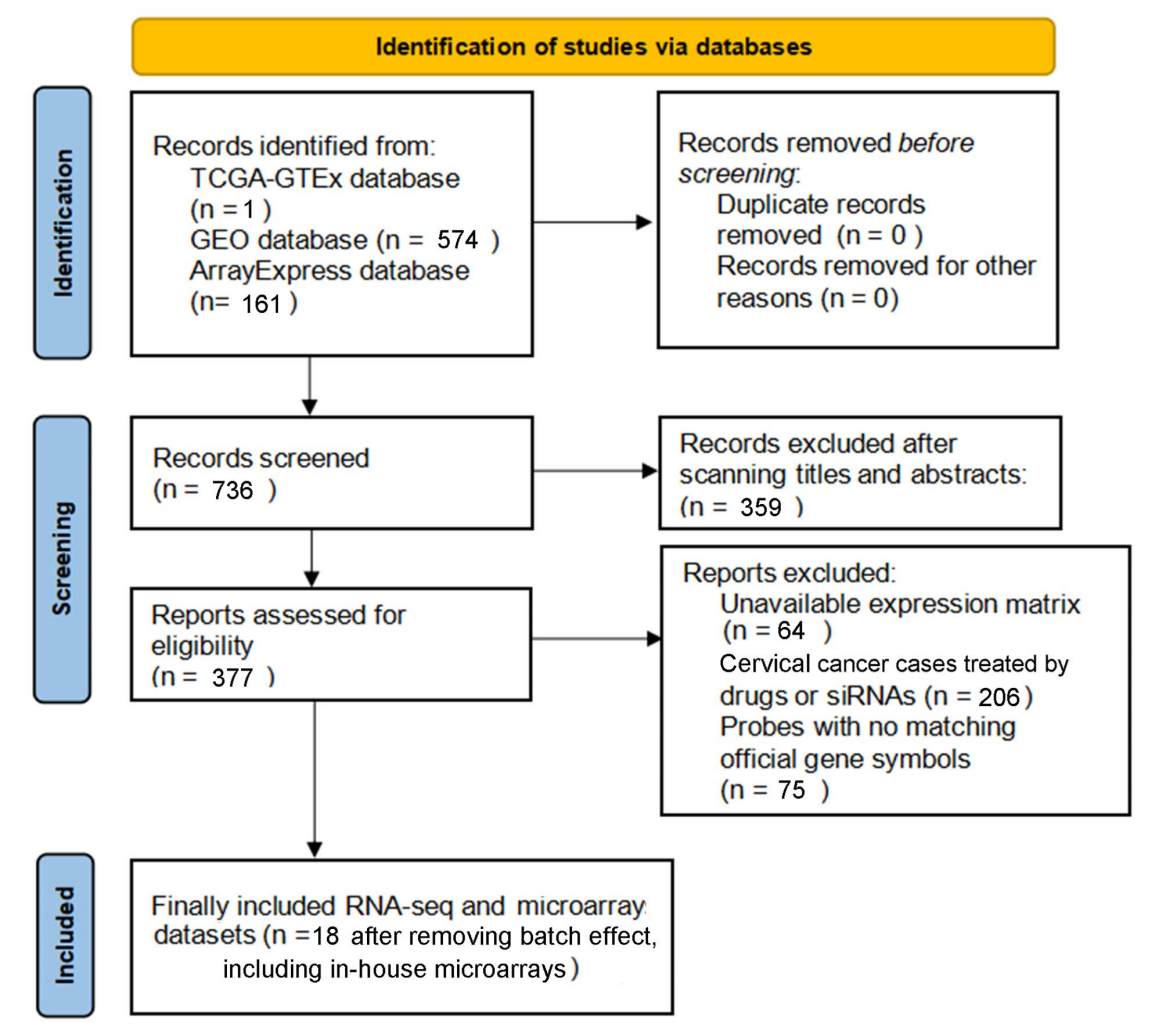
Flow chart of data collection for this study

**Fig. 4:**
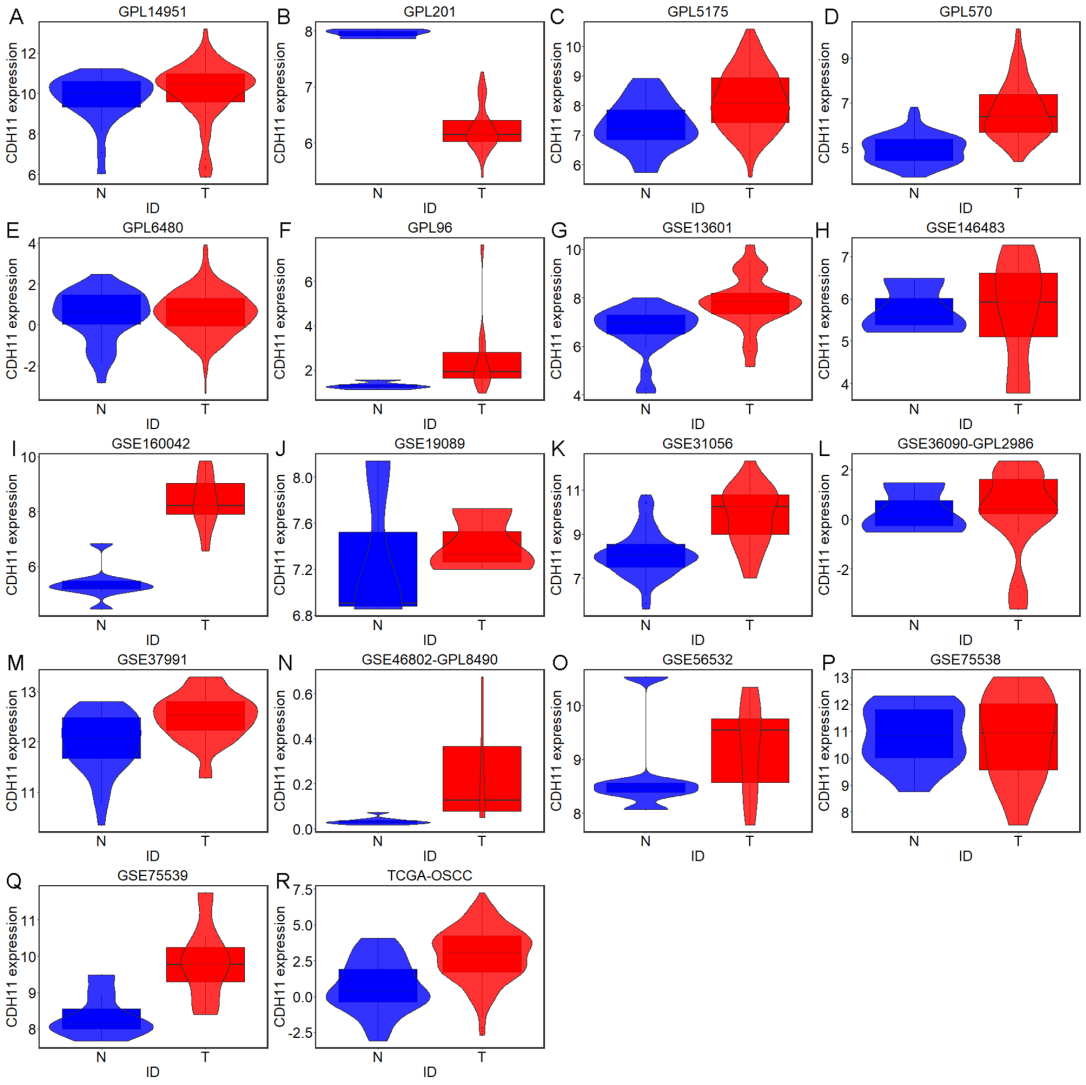
Violin plots of CDH11 expression between oral squamous cell carcinoma and non-cancer oral specimen in per studies. (A) GPL14951. (B) GPL201. (C) GPL5175. (D) GPL570. (E) GPL6480. (F) GPL96. (G) GSE13601. (H) GSE146483. (I) GSE160042. (J) GSE19089. (K) GSE31056. (L) GSE36090-GPL2986. (M) GSE37991. (N) GSE46802-GPL8490. (O) GSE56532. (P) GSE75538. (Q) GSE75539. (R) TCGA – oral squamous cell carcinoma. N, non-cancer oral specimen; T, tumor specimen

**Fig. 5:**
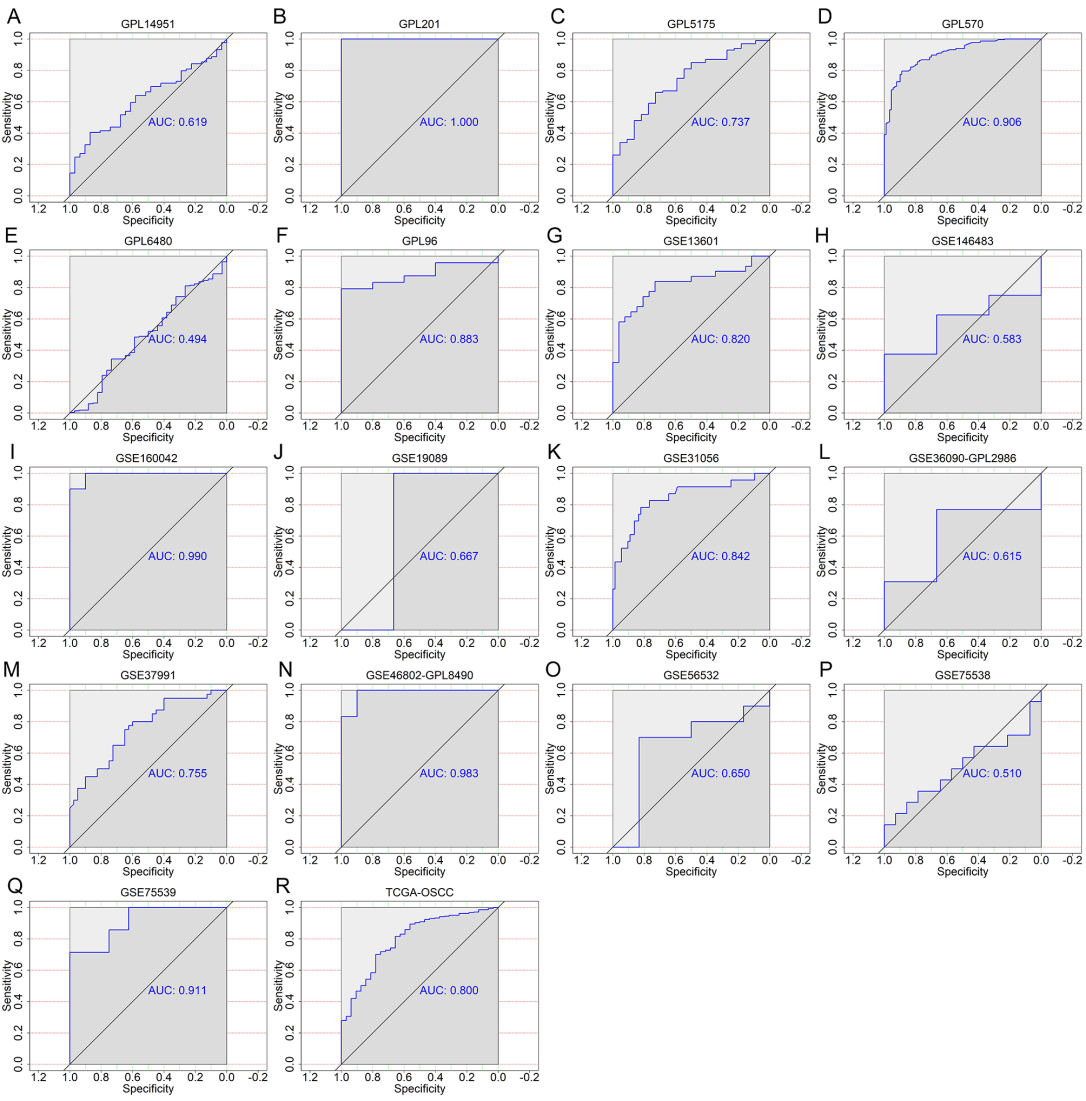
ROC curves indicating the ability of the differential expression of CDH11 to distinguish oral squamous cell carcinoma tissues from non-cancerous tissues in per studies. (A) GPL14951. (B) GPL201. (C) GPL5175. (D) GPL570. (E) GPL6480. (F) GPL96. (G) GSE13601. (H) GSE146483. (I) GSE160042. (J) GSE19089. (K) GSE31056. (L) GSE36090-GPL2986. (M) GSE37991. (N) GSE46802-GPL8490. (O) GSE56532. (P) GSE75538. (Q) GSE75539. (R) TCGA – oral squamous cell carcinoma. AUC, area under the curve

**Fig. 6:**
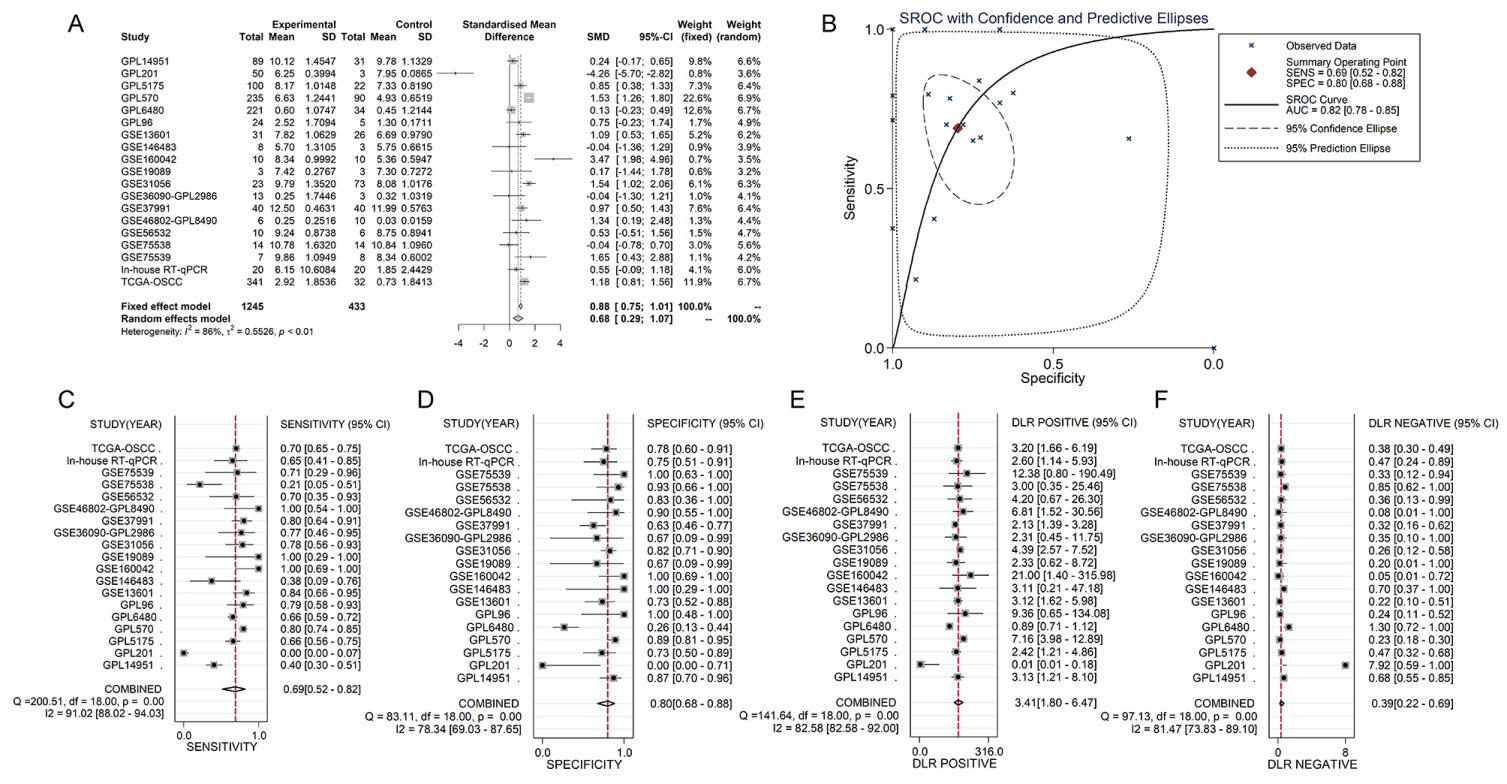
Overall evaluation of CDH11 expression and the ability of the differential expression of CDH11 to distinguish oral squamous cell carcinoma tissues from non-cancerous tissues. (A) The pooled level of CDH11 expression between the oral squamous cell carcinoma tissues and non-cancerous tissues. (B) Summarized ROC curve; AUC, area under the curve. (C) Pooled forest plots of sensitivity. (D) Pooled forest plots of specificity. (E) Pooled forest plots of diagnostic likelihood ratio positive. (F) Pooled forest plots of diagnostic likelihood ratio negative; DLR, diagnostic likelihood ratio

**Fig. 7:**
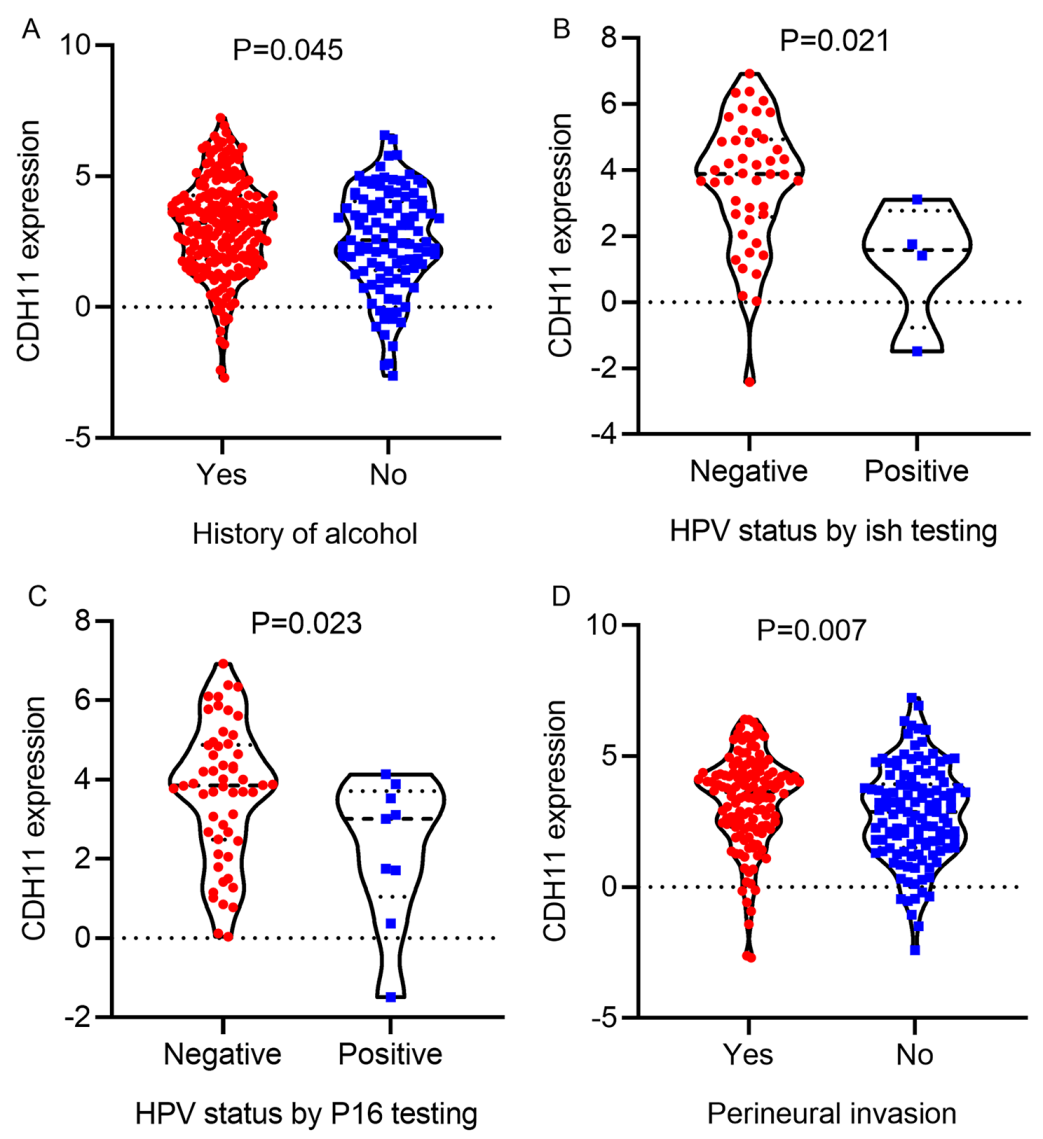
Relationships between CDH11 expression and the clinico-pathological features of oral squamous cell carcinoma patients from RNA-seq dataset. (A) The expression of CDH11 in different patient groups by alcohol history. (B) The expression of CDH11 in different patient groups of HPV status by ISH testing. (C) The expression of CDH11 in different patient groups of HPV status by P16 testing. (D) The expression of CDH11 in different patient groups by perineural invasion

The TCGA-GTEx RNA-seq dataset and 17 microarrays processed after the removal of batch effects were analyzed via differential expression analysis (Table S1). The procedures involved in selecting appropriate the RNS-seq dataset and microarrays for the current study are exhibited in Fig. 3. It can be seen from the violin plots and ROC curves that the CDH11 expression was obviously higher in the OSCC than in the non-cancer oral specimen in most of the RNA-seq dataset and microarrays, and overexpressed CDH11 exhibited preferable performance in distinguishing between the OSCC and non-cancer oral samples (Figs. 4 and 5). The overall forest plot of the SMD and SROC curves generated through pooling the expression data of CDH11 in the OSCC and non-cancer oral samples from all the included datasets confirmed the significant overexpression of CDH11 in the OSCC tissues and the preferable discriminatory ability of CDH11 expression for OSCC (SMD = 0.88, 95%CI = 0.75–1.01) (AUC = 0.82, 95%CI = 0.78–0.85) (Fig. 6). With regard to the relationship between CDH11 expression and the clinico-pathological features of the OSCC patients from the RNA-seq dataset, the up-regulation of CDH11 was remarkably associated with a history of alcohol, negative HPV status (determined via ISH testing), negative HPV status (determined via P16 testing), and perineural invasion of the OSCC patients (p < 0.05) (Fig. 7).

### The effect of CDH11 expression on the overall survival of OSCC patients

**Fig. 8:**
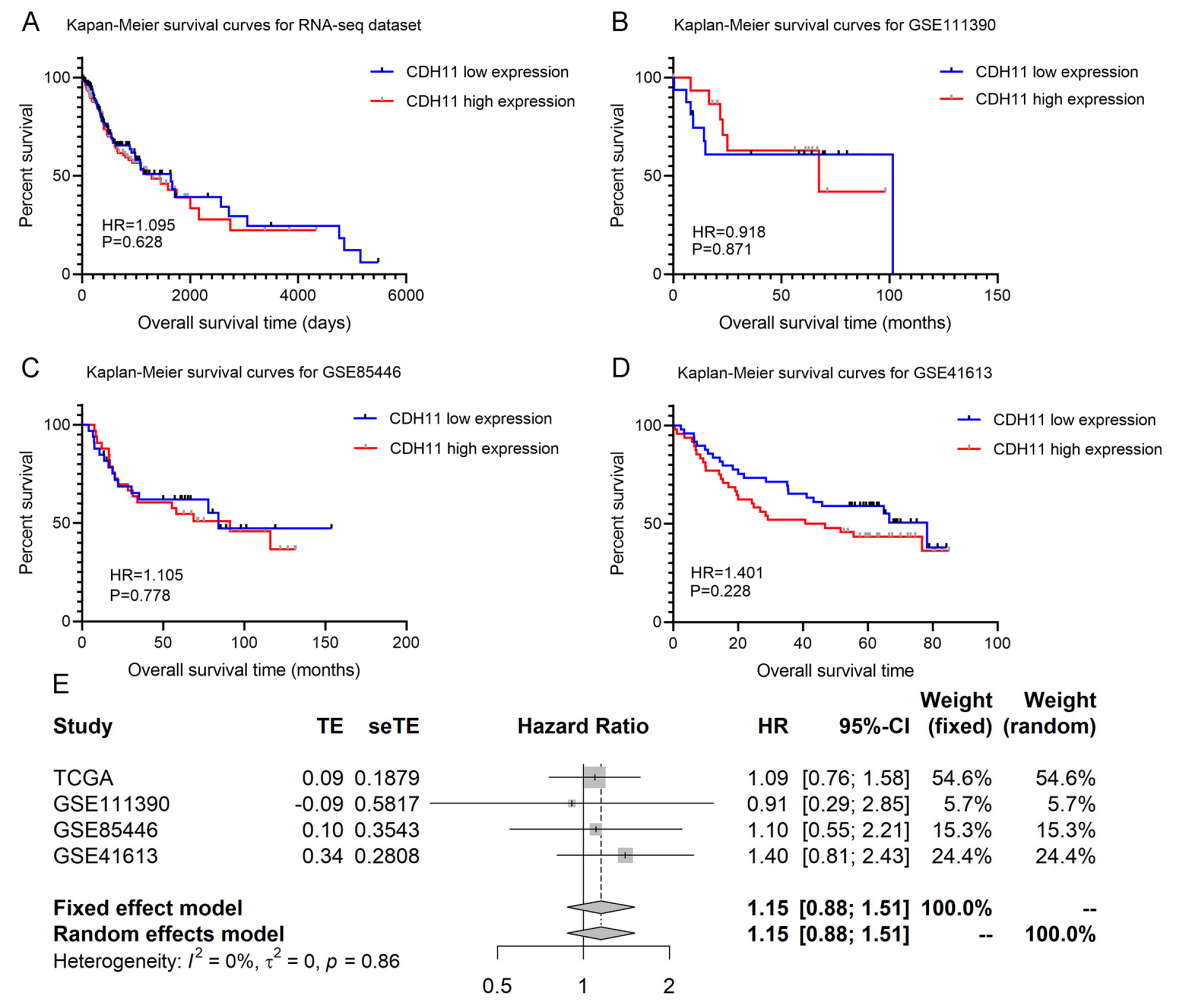
Prognosis value of CDH11 in oral squamous cell carcinoma. (A) Kaplan-Meier survival curves based on TCGA database. (B) Kaplan-Meier survival curves based on GSE111390. (C) Kaplan-Meier survival curves based on GSE85446. (D) Kaplan-Meier survival curves based on GSE41613. (E) Forest plot combining all datasets. HR, hazard ratio; TE, logarithms hazard ratio

The prognostic value of CDH11 expression in predicting the overall survival outcome of the OSCC patients was analyzed in four cohorts from the GSE111390, GSE85446, GSE41613, and TCGA database. The overall survival difference between the OSCC patients with high versus low CDH11 expression—reflected by Kaplan-Meier survival curves—was insignificant. A forest plot of the HR values showed a vague trend that higher CDH11 expression might serve as a risk factor for the overall survival of OSCC patients (HR = 1.15, 0.88–1.51) (Fig. 8).

### Predicted transcription factors and miRNAs of CDH11 in OSCC

To better understand the upstream transcriptional regulation mechanism of CDH11 in OSCC, a transcription factor/miRNA-mRNA network was built for seeking possible transcription factors or miRNAs that modulate the expression of CDH11. It can be inferred from the network that CDH11 was connected to multiple transcription factors and miRNAs, such as CTNNB1, NFATC2, CEBPA, miR-376c, and miR-205 (Figure S3).

### Relationship between CDH11 expression and infiltration level of immune cells in OSCC

**Fig. 9:**
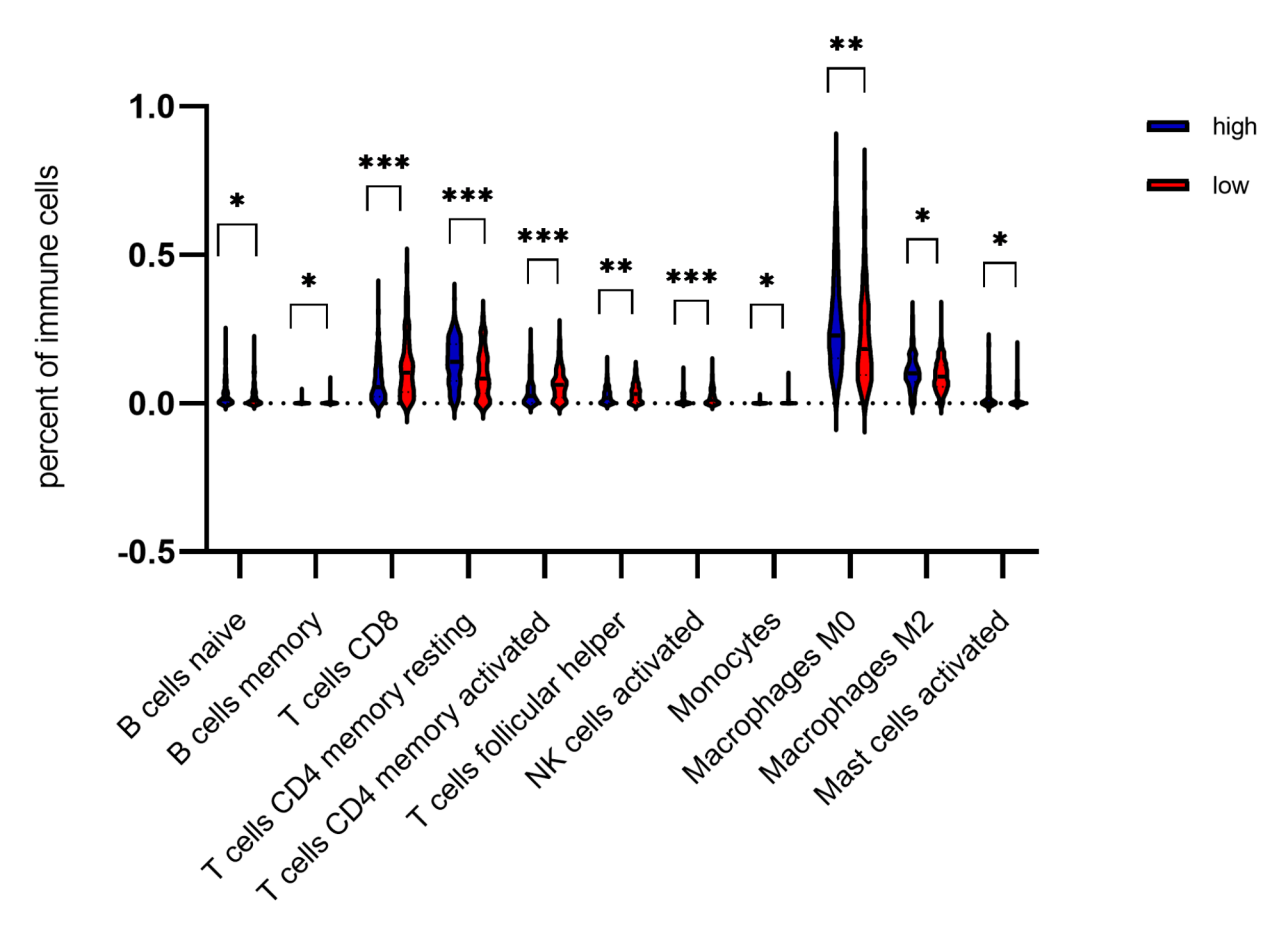
Relationship between CDH11 expression and infiltration level of immune cells in oral squamous cell carcinoma. * p < 0.05; **p < 0.01, ***p < 0.001

The RNA-seq dataset from the TCGA database was chosen for immune correlation analysis, as it has the largest sample size among all the included datasets. Naïve B cells naïve, resting CD4 memory T cells, M1 macrophages, M2 macrophages, and activated mast cells were found to have higher infiltration levels in the OSCC patients with higher CDH11 expression, while memory B cells, CD8 T cells, activated CD4 memory T cells, follicular helper T cells, activated NK cells, and monocytes were found have a higher proportion in the OSCC patients with lower CDH11 expression (p < 0.05) (Table 2). The violin plot in Fig. 9 displays part of the significantly different distributions of the immune cells in the OSCC patients with low versus high CDH11 expression.

**Table 2:** Distribution of the proportions of different immune cells in OSCC patients with high or low CDH11 expression

Types of immune cells	Subgroup	Proportions of immune cells in low or high CDH11 expression groups			t	P-value
		Number	Mean	Standard deviation		
B cells naive	CDH11 low expression group	170	0.025	0.035	2.045	0.042
	CDH11 high expression group	171	0.034	0.046		
B cells memory	CDH11 low expression group	170	0.003	0.010	-2.039	0.042
	CDH11 high expression group	171	0.001	0.005		
T cells CD8	CDH11 low expression group	170	0.118	0.099	-4.661	< 0.001
	CDH11 high expression group	171	0.075	0.069		
T cells CD4 memory resting	CDH11 low expression group	170	0.098	0.079	4.681	< 0.001
	CDH11 high expression group	171	0.138	0.076		
T cells CD4 memory activated	CDH11 low expression group	170	0.065	0.053	-4.152	< 0.001
	CDH11 high expression group	171	0.043	0.048		
T cells follicular helper	CDH11 low expression group	170	0.035	0.030	-2.919	0.004
	CDH11 high expression group	171	0.026	0.027		
NK cells activated	CDH11 low expression group	170	0.023	0.029	-4.492	< 0.001
	CDH11 high expression group	171	0.011	0.018		
Monocytes	CDH11 low expression group	170	0.004	0.013	-2.105	0.036
	CDH11 high expression group	171	0.002	0.005		
Macrophages M0	CDH11 low expression group	170	0.215	0.148	3.283	0.001
	CDH11 high expression group	171	0.269	0.159		
Macrophages M2	CDH11 low expression group	170	0.093	0.051	2.111	0.035
	CDH11 high expression group	171	0.106	0.060		
Mast cells activated	CDH11 low expression group	170	0.020	0.035	2.333	0.02
	CDH11 high expression group	171	0.031	0.050		

Note: Only significant differential distribution (P < 0.05) was displayed in the Table

### Molecular functions of CDH11-related genes in OSCC

A total of 3,469 significantly upregulated genes and 2,639 significantly downregulated genes with consistent SMD values were identified as the final DEGs of OSCC (Table S2). Of the 3,469 identified up-regulated DEGs and 2,639 identified down-regulated DEGs in OSCC, 209 genes positively co-expressed with CDH11 and 147 genes negatively co-expressed with CDH11 were screened out (Figure S4). Genes positively co-expressed with CDH11 were annotated to cluster in biological processes and pathways, including extracellular matrix organization, extracellular structure organization, protein digestion and absorption, and ECM-receptor interaction (Figure S5). Other biological processes and pathways, such as epidermal cell differentiation, the estrogen metabolic process, steroid hormone biosynthesis, and retinol metabolism, were prominently assembled by genes negatively co-expressed with CDH11 (Figure S6).

### Whole-genome sequencing of mouse oral squamous cell carcinoma model

The single nucleotide variants (SNVs) of the tumor cell exon regions in all parts of the nine mice were extracted separately, including mouse tongue, submandibular lymph nodes, bone marrow, normal muscle tissues pecimens, and the SNVs related to tumors were screened with reference to the Catalogue of Somatic Mutations in Cancer (COSMIC) database. A total of 238 tumor-related SNVs and 120 high-frequency mutation genes with a mutation frequency ≥ 5% ( Table S3) were obtained via whole-genome sequencing of 36 samples of the nine mice. Sirpb1a, Cdh11, Smarca4, Fat1, Gata3, Notch1, Usp32, Cdk12, Cic, and Creb3l2 were the top 10 highly mutated genes.

### Single-cell functional analysis

**Fig. 10:**
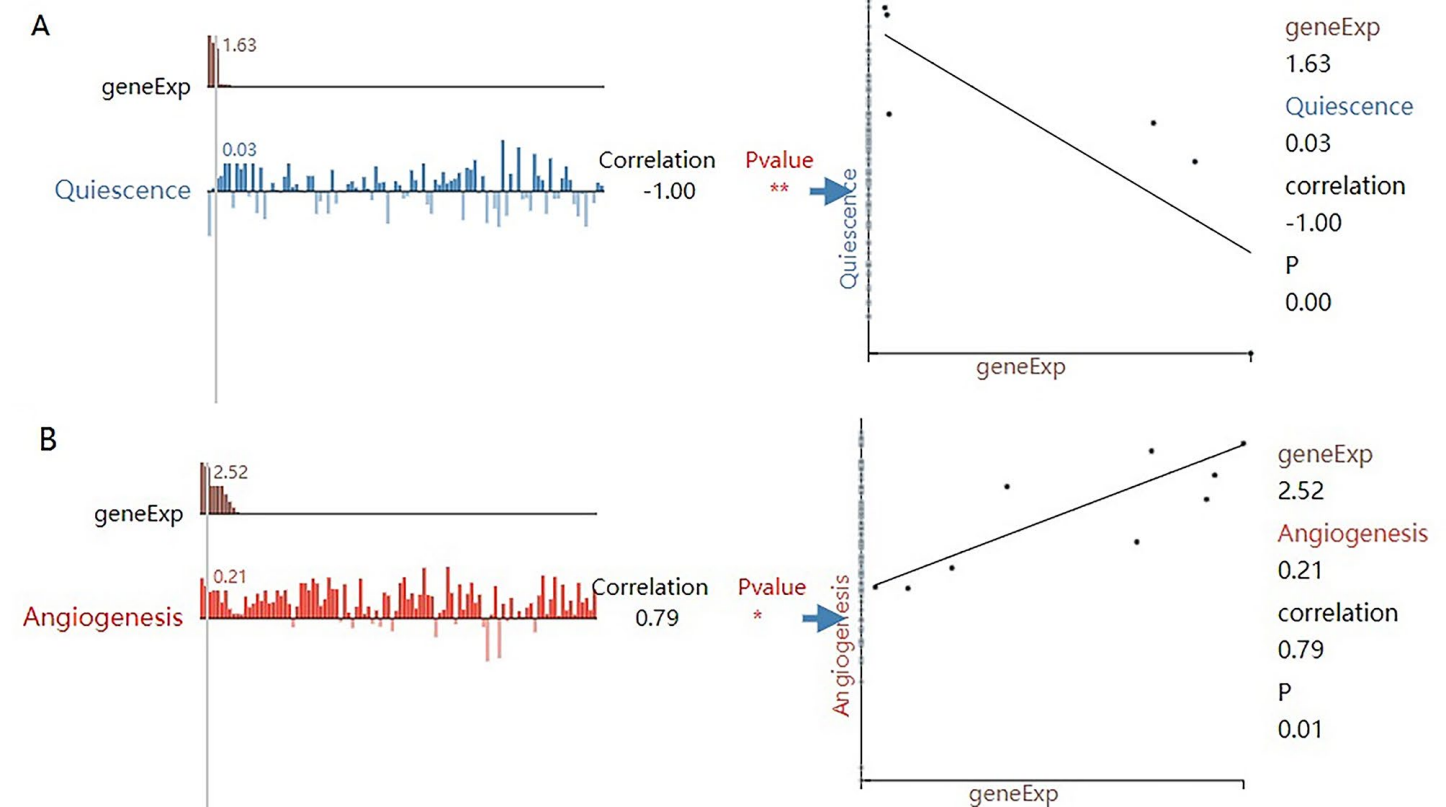
Functional relevance in different head and neck cancer cell groups with CDH11 from CancerSEA. (A) Correlation between CDH11 expression and functional states in MEEI26 cell group. (B) Correlation between CDH11 expression and functional states in MEEI5 cell group. In the scatter plot, the x-axis indicates the expression of CDH11, and the y-axis indicates the activity of the functional state.* p < 0.05; **p < 0.01

Figure 10 shows the correlations between CDH11 and the functional states in a cell subpopulation of head and neck cancer cells. CDH11 expression was negatively correlated with the quiescence of the MEEI26 cell group in HNSCC (Fig. 10A) (Spearman’s coefficient = -1.00, p < 0.01). Also, CDH11 expression was positively correlated with the angiogenesis of the MEEI5 cell group in HNSCC (Fig. 10B) (Spearman’s coefficient = 0.79, p < 0.05).

## Discussion

At present, genome research has entered the era of the functional genome—that is, the post-genome era. In the post-genome era, it is necessary not only to understand the base sequence of the genome but also to study the genome function and explore the mystery of human health and disease at the overall molecular level [15]. At present, research projects on functional genomes, proteomes, and drug genomes are booming [16]. In the face of the massive data generated by such studies, it is useless to rely solely on traditional experimental observation methods. We must also rely on high-performance computers and efficient data processing algorithm language, which establishes the fundamental status of computational biology in the post-genome era [17].

The occurrence and development of OSCC is an extremely complex process with intricate biological behaviors. Because of the high incidence and poor prognosis of OSCC, the study of its pathogenesis is particularly important. Currently, OSCC is considered to be caused by the abnormal expression of diverse oncogenes or tumor-suppressor genes and signal transduction molecules [18]. Herein, we focused on CDH11 to investigate the clinico-pathological significance and molecular basis of CDH11 in OSCC through a combination of RT-qPRC, western blot, IHC, whole-genome sequencing, and computational biology.

This is the first study evaluating the expression significance of CDH11 in OSCC and non-cancer oral samples via various detection methods. The results from the in-house RT-qPCR, western blot, and IHC consistently supported the overexpression of CDH11 in OSCC tissues compared with non-cancer oral samples; these findings were further corroborated by differential expression analysis of globally gathered multi-center large samples (1225 OSCC samples and 413 non-cancer oral samples), ensuring the credibility of the analysis results in the present study. Association analysis with the clinical parameters of the in-house OSCC patients and other cohorts revealed significant relationships between high CDH11 expression and the malignant progression of OSCC patients manifested by lymph node metastasis and perineural infiltration. The perineural invasion of OSCC is closely linked with lymph node metastasis, which means the high invasiveness of OSCC [19]. The above results implied that OSCC patients with a high expression of CDH11 may be at an increased risk when than compared with OSCC patients with a low expression of CDH11. It was assumed that CDH11 overexpression in OSCC might guide the prognostic stratification or post-operation radiotherapy of OSCC patients. The promotive effect of CDH11 expression in the metastasis of human cancers has been discovered in prostate cancer and early luminal breast cancer [5, 20]. Bonneau et al. found that the high expression of CDH11 mediated the pro-metastatic activity of a particular subset of cancer-associated fibroblasts in early luminal breast cancer [20], from which we posit that the influence of CDH11 expression on the propensity for metastasis in OSCC patients might be relevant to cancer-associated fibroblasts.

After comprehensive evaluation of the clinico-pathological value of CDH11 in OSCC, the scientific basis of CDH11 in OSCC was further investigated via prediction of the upstream transcription network, immune correlation analysis, and functional annotations of genes co-expressed with CDH11. The CDH11-centered miRNA/TF transcriptional network revealed potential regulators underlying the up-regulation of CDH11 in OSCC. The tumor microenvironment (TME) consists of all the non-cancerous host cells and the non-cellular components in the tumor [21]. Among them, immune cells represent a large fraction of the tumor microenvironment. TME is a critical regulator of tumor growth, progression and metastasis [22]. Loss or mutation of p53 in cancers can function in immune cells, allowing immune evasion and promoting cancer progression [23]. BCL9/BCL9L inhibited the infiltration of CD8 + T cells in the tumor microenvironment, and promoted striple-negative breast cancer growth through the Wnt and TGF-βpathways [24]. High RACK1 expression in OSCC cells correlated with increased M2 macrophage infiltration, and promoted cancer progression by increasing the M2/M1 macrophage ratio via the NF-κB pathway [25]. There is growing evidence supporting TME in tumor formation and progression. Consequently, from the intricate relationships between CDH11 expression and the infiltration of multiple immune cells, we speculate that the interactions between the tumor microenvironment and CDH11 played key roles in the oncogenic influence of CDH11 in OSCC. We also paid attention to genes co-expressed with CDH11 in OSCC and extended the analysis to the biological process, molecular functions, and pathways enriched by genes positively or negatively co-expressed with CDH11 in OSCC. The majority of the biological processes or pathways that were significantly clustered by co-expressed genes, including extracellular matrix organization, the epithelial to mesenchymal transition, carbon metabolism, and the PI3K-Akt signaling pathway, have been reported to be involved in the carcinogenesis of OSCC [26–39]. Thus, CDH11 might contribute to the occurrence and development of OSCC through co-work with co-expressed genes participating in these altered biological processes or pathways. Furthermore, cancer is a genetic disease due to the accumulation of numerous mutations. Single-nucleotide variants (SNVs) are the most common genetic variants playing a significant role in the occurrence and development of cancer [40]. Based on the Whole-genome sequencing of mouse oral squamous cell carcinoma model, CDH11 was found to be a high-frequency mutation gene in OSCC, which suggests its importance in the progression of OSCC. Tumor angiogenesis is critical for tumors, including HNSCC progression, as the new blood vessels supply nutrients and facilitate metastasis [41, 42]. Quiescence is essential for cancer cells to acquire additional mutations, survive in a new environment and initiate metastasis, become resistant to cancer therapy, and evade immune destruction [43]. CDH11 expression was correlated with angiogenesis and quiescence in a cell subpopulation of head and neck cancer cells, suggestting the importance of CDH11 during carcinogensis by regulating these two single-cell functional states.

The limitations of the present study include its lack of experimental validation of the functional roles of CDH11 in OSCC, the detection of CDH11 mRNA and protein expression in mouse OSCC model, and the connections between CDH11 and other co-expressed genes; these should thus be focuses of future work.

## Conclusions

In summary, CDH11 was identified to be overexpressed in OSCC and related to its clinical progression. The oncogenic influence of CDH11 overexpression in OSCC was related to the infiltration of immune cells and pathways, including ECM-receptor interaction, carbon metabolism, and the PI3K-Akt signaling pathway. Additionally, that CDH11 was highly mutated in various stages of OSCC by whole-genome sequencing revealed the genomic changes of CDH11 in OSCC. Overall, CDH11 might serve as a valuable biomarker in OSCC.

## Electronic supplementary material

Below is the link to the electronic supplementary material.

Supplementary Material 1

Supplementary Material 2

Supplementary Material 3

Supplementary Material 4

## Data Availability

The data generated and analyzed during the current study was uploaded and now is available in the Sequence Read Archive (SRA) database (PRJNA837417, https://www.ncbi.nlm.nih.gov/sra/PRJNA837417).

## Abbreviations

TCGA: The cancer genome atlas
HPV: Human papilloma virus
GEO: Gene expression omnibus
RIPA: Radio immunoprecipitation assay
HRP: Horseradish peroxidase
FPKM: Fragments per kilobase per million
SMD: Standard mean deviation
SROC: Summarized receiver’s operating characteristics
HR: hazard ratio
CI: Confidence interval
M: Mean
SD: Standard deviation
ANOVA: Analysis of variance
DEGs: Differentially expressed genes
4-NQO: 4-nitroquinoline-1-oxide
MACS: Magnetic activated cell sorting
DTCs: Disseminated tumor cells
HNSCC: Head and neck squamous cell carcinoma
PNI+: Perineural infiltration
PNI-: Perineural infiltration (PNI-)
SNVs: Single nucleotide variants
COSMIC: Catalogue of somatic mutations in cancer.
