# Supplementary material for: Expression signature and molecular basis of CDH11 in OSCC detected by a combination of multiple methods
Source: BMC Med Genomics. 2023 Apr 3;16:70. doi: 10.1186/s12920-023-01499-7 (PMC10069064; doi:10.1186/s12920-023-01499-7)
Supplement: Supplementary file 1 — Supplementary Material 1 [file 12920_2023_1499_MOESM1_ESM.docx]

Supplementary Table 1. Basic elements of all included microarrays and RNA-seq datasets for differential expression analysis

| Platform | Included microarrays | Year | | Number of samples | | | | Experiment type | | Contact Author | | Country | |  |
| --- | --- | --- | --- | --- | --- | --- | --- | --- | --- | --- | --- | --- | --- | --- |
|  |  |  |  | OSCC tissues | | Non-cancer oral tissues | |  |  |  |  |  |  |  |
| GPL14951 | GSE34105 | 2012 | | 89 | | 31 | | Expression profiling by array | | Matilda Rentoft | | Sweden | |  |
|  | GSE34106 | 2012 | |  |  |  |  | Expression profiling by array | | Matilda Rentoft | | Sweden | |  |
| GPL201 | GSE21866 | 2014 | | 50 | | 3 | | Expression profiling by array | | Roland Grafström | | Sweden | |  |
|  | GSE51010 | 2014 | |  |  |  |  | Expression profiling by array | | anas amjad saeed | | United Kingdom | |  |
| GPL570 | GSE30784 | 2011 | | 235 | | 90 | | Expression profiling by array | | Chu Chen | | USA | |  |
|  | GSE31853 | 2011 | |  |  |  |  | Expression profiling by array | | Wenbin Wei | | United Kingdom | |  |
|  | GSE51010 | 2014 | |  |  |  |  | Expression profiling by array | | anas amjad saeed | | United Kingdom | |  |
|  | GSE74530 | 2017 | |  |  |  |  | Expression profiling by array | | Steve Oghumu | | USA | |  |
|  | GSE78060 | 2017 | |  |  |  |  | Expression profiling by array | | Tomohiro Enokida | | Japan | |  |
|  | GSE9844 | 2008 | |  |  |  |  | Expression profiling by array | | Tianwei Yu | | USA | |  |
|  | GSE98942 | 2018 | |  |  |  |  | Expression profiling by array | | Xiaofeng Zhou | | USA | |  |
|  | GSE43862 | 2013 | |  |  |  |  | Expression profiling by array | | Yoshiaki Tabuchi | | Japan | |  |
|  | GSE138206 | 2019 | |  |  |  |  | Expression profiling by array | | Hao Pan | | China | |  |
| GPL96 | GSE3524 | 2005 | | 24 | | 5 | | Expression profiling by array | | Gokce Toruner | | USA | |  |
|  | GSE31853 | 2011 | |  |  |  |  | Expression profiling by array | | Wenbin Wei | | United Kingdom | |  |
| GPL5175 | GSE25099 | 2011 | | 100 | | 22 | | Expression profiling by array | | Chien-Hua Peng | | Taiwan | |  |
|  | GSE41116 | 2013 | |  |  |  |  | Expression profiling by array | | Jianjun Zhang | | USA | |  |
| GPL6480 | GSE84846 | 2017 | | 221 | | 34 | | Expression profiling by array | | Nicoletta Bertani | | Italy | |  |
|  | GSE85446 | 2017 | |  |  |  |  | Expression profiling by array | | Daoud Sie | | Netherlands | |  |
|  | GSE23558 | 2011 | |  |  |  |  | Expression profiling by array | | Manoj Balkrishna Mahimkar | | India | |  |
|  | GSE142583 | 2021 | |  |  |  |  | Expression profiling by array | | Paola Ostano | | Italy | |  |
| GPL8300 | GSE13601 | 2008 | | 31 | | 26 | | Expression profiling by array | | Nicholas D Socci | | USA | |  |
| GPL6947 | GSE19089 | 2009 | | 3 | | 3 | | Expression profiling by array | | Yan W. Asmann | | USA | |  |
| GPL10526 | GSE31056 | 2011 | | 23 | | 73 | | Expression profiling by array | | Levi Waldron | | USA | |  |
| GPL2986 | GSE36090 | 2012 | | 13 | | 3 | | Expression profiling by array | | Koh-ichi Nakashiro | | Japan | |  |
| GPL6883 | GSE37991 | 2013 | | 40 | | 40 | | Expression profiling by array | | Chia Huei Lee | | Taiwan | |  |
| GPL8490 | GSE46802 | | 2013 | | 6 | | 10 | | Expression profiling by array | | Rebecca M Towle | | Canada | |
| GPL10739 | GSE56532 | 2014 | | 10 | | 6 | | Expression profiling by array | | sivapriya pavuluri | | Australia | |  |
| GPL18281 | GSE75538 | 2016 | | 14 | | 14 | | Expression profiling by array | | binay panda | | India | |  |
| GPL18282 | GSE75539 | 2016 | | 7 | | 8 | | Expression profiling by array | | binay panda | | India | |  |
| GPL17077 | GSE146483 | 2020 | | 8 | | 3 | | Expression profiling by array | | Yutaro Kase | | Japan | |  |
| GPL18180 | GSE160042 | 2021 | | 10 | | 10 | | Expression profiling by array | | Zehang Zhuang | | China | |  |
| TCGA | NA | NA | | 296 | | 30 | | RNA-sequencing | | -- | | -- | |  |
