# Supplementary material for: Expression signature and molecular basis of CDH11 in OSCC detected by a combination of multiple methods
Source: BMC Med Genomics. 2023 Apr 3;16:70. doi: 10.1186/s12920-023-01499-7 (PMC10069064; doi:10.1186/s12920-023-01499-7)
Supplement: Supplementary file 2 — Supplementary Material 2 [file 12920_2023_1499_MOESM2_ESM.docx]

Supplementary Table 3. High-frequency mutation genes with mutation frequency（≥5%）obtained by whole-genome sequencing

| Gene | Mutations count | Mutation probability | Mutations cases | | |
| --- | --- | --- | --- | --- | --- |
| Sirpb1a | 10 | 37.04 | ['LNDTCs2', 'pri1', 'BMDTCs1', 'LNDTCs1', 'pri7', 'BMDTCs7', 'LNDTCs7', 'pri6', 'BMDTCs6', 'LNDTCs6'] | | |
| Cdh11 | 8 | 29.63 | ['pri1', 'BMDTCs1', 'LNDTCs1', 'pri4', 'BMDTCs4', 'LNDTCs4', 'BMDTCs3', 'BMDTCs6'] | | |
| Smarca4 | 7 | 25.93 | ['pri2', 'pri6', 'BMDTCs6', 'LNDTCs6', 'BMDTCs9', 'LNDTCs9', 'LNDTCs8'] | | |
| Fat1 | 5 | 18.52 | ['LNDTCs2', 'LNDTCs4', 'LNDTCs3', 'BMDTCs8', 'LNDTCs8'] | | |
| Gata3 | 5 | 18.52 | ['LNDTCs1', 'BMDTCs9', 'LNDTCs9', 'BMDTCs8', 'LNDTCs8'] | | |
| Notch1 | 5 | 18.52 | ['BMDTCs6', 'BMDTCs9', 'LNDTCs9', 'BMDTCs8', 'LNDTCs8'] | | |
| Usp32 | 5 | 18.52 | ['BMDTCs2', 'LNDTCs6', 'BMDTCs9', 'BMDTCs8', 'LNDTCs8'] | | |
| Cdk12 | 4 | 14.81 | ['BMDTCs9', 'LNDTCs9', 'BMDTCs8', 'LNDTCs8'] | | |
| Cic | 4 | 14.81 | ['BMDTCs7', 'BMDTCs9', 'LNDTCs9', 'BMDTCs8'] | | |
| Creb3l2 | 4 | 14.81 | ['LNDTCs1', 'pri7', 'BMDTCs7', 'LNDTCs7'] | | |
| Csmd3 | 4 | 14.81 | ['pri2', 'pri7', 'LNDTCs3', 'BMDTCs8'] | | |
| Hsp90ab1 | 4 | 14.81 | ['pri5', 'BMDTCs5', 'LNDTCs5', 'LNDTCs3'] | | |
| Kmt2d | 4 | 14.81 | ['pri7', 'LNDTCs3', 'BMDTCs6', 'LNDTCs8'] | | |
| Mycl | 4 | 14.81 | ['BMDTCs9', 'LNDTCs9', 'BMDTCs8', 'LNDTCs8'] | | |
| Myod1 | 4 | 14.81 | ['BMDTCs9', 'LNDTCs9', 'BMDTCs8', 'LNDTCs8'] | | |
| Ptpn13 | 4 | 14.81 | ['LNDTCs2', 'pri7', 'LNDTCs4', 'LNDTCs3'] | | |
| Ranbp2 | 4 | 14.81 | ['LNDTCs2', 'BMDTCs9', 'LNDTCs9', 'LNDTCs8'] | | |
| Ros1 | 4 | 14.81 | ['pri2', 'BMDTCs1', 'pri7', 'pri4'] | | |
| Trrap | 4 | 14.81 | ['LNDTCs7', 'BMDTCs9', 'LNDTCs9', 'LNDTCs8'] | | |
| Wnk2 | 4 | 14.81 | ['BMDTCs2', 'pri4', 'BMDTCs8', 'LNDTCs8'] | | |
| Znrf3 | 4 | 14.81 | ['BMDTCs9', 'LNDTCs9', 'BMDTCs8', 'LNDTCs8'] | | |
| Arhgef12 | 3 | 11.11 | ['BMDTCs2', 'pri7', 'BMDTCs6'] | | |
| Bcl11b | 3 | 11.11 | ['BMDTCs9', 'LNDTCs9', 'BMDTCs8'] | | |
| Bcl9 | 3 | 11.11 | ['pri2', 'LNDTCs4', 'LNDTCs8'] | | |
| Brca2 | 3 | 11.11 | ['pri2', 'BMDTCs1', 'BMDTCs8'] | | |
| Cdh1 | 3 | 11.11 | ['BMDTCs7', 'LNDTCs6', 'LNDTCs8'] | | |
| Cdh17 | 3 | 11.11 | ['pri2', 'BMDTCs9', 'LNDTCs8'] | | |
| Erc1 | 3 | 11.11 | ['LNDTCs4', 'BMDTCs9', 'LNDTCs9'] | | |
| Fat4 | 3 | 11.11 | ['pri2', 'pri6', 'LNDTCs8'] | | |
| Fbln2 | 3 | 11.11 | ['pri4', 'BMDTCs9', 'LNDTCs9'] | | |
| Foxa1 | 3 | 11.11 | ['pri7', 'BMDTCs8', 'LNDTCs8'] | | |
| Grm3 | 3 | 11.11 | ['BMDTCs9', 'LNDTCs9', 'LNDTCs8'] | | |
| Jazf1 | 3 | 11.11 | ['BMDTCs7', 'BMDTCs9', 'LNDTCs9'] | | |
| Lrp1b | 3 | 11.11 | ['pri7', 'BMDTCs6', 'BMDTCs8'] | | |
| Mpl | 3 | 11.11 | ['pri2', 'LNDTCs1', 'pri3'] | | |
| Muc4 | 3 | 11.11 | ['LNDTCs2', 'LNDTCs1', 'pri7'] | | |
| Myh11 | 3 | 11.11 | ['pri2', 'LNDTCs4', 'pri6'] | | |
| Ncoa2 | 3 | 11.11 | ['pri7', 'BMDTCs9', 'LNDTCs9'] | | |
| Nfatc2 | 3 | 11.11 | ['BMDTCs9', 'LNDTCs9', 'LNDTCs8'] | | |
| Polg | 3 | 11.11 | ['BMDTCs2', 'BMDTCs9', 'LNDTCs9'] | | |
| Prex2 | 3 | 11.11 | ['LNDTCs2', 'BMDTCs7', 'LNDTCs3'] | | |
| Rsl1 | 3 | 11.11 | ['pri1', 'BMDTCs1', 'LNDTCs1'] | | |
| Setbp1 | 3 | 11.11 | ['LNDTCs4', 'LNDTCs3', 'LNDTCs8'] | | |
| Setd1b | 3 | 11.11 | ['pri7', 'BMDTCs9', 'LNDTCs9'] | | |
| Spen | 3 | 11.11 | ['pri4', 'LNDTCs6', 'BMDTCs9'] | | |
| Srgap3 | 3 | 11.11 | ['pri2', 'BMDTCs7', 'LNDTCs6'] | | |
| Tet2 | 3 | 11.11 | ['BMDTCs2', 'BMDTCs1', 'pri7'] | | |
| Thrap3 | 3 | 11.11 | ['pri9', 'BMDTCs9', 'LNDTCs9'] | | |
| Trim24 | 3 | 11.11 | ['pri7', 'BMDTCs6', 'BMDTCs8'] | | |
| Tshr | 3 | 11.11 | ['pri2', 'BMDTCs6', 'LNDTCs8'] | | |
| Abi1 | 2 | 7.41 | ['LNDTCs1', 'LNDTCs8'] | |  |
| Abl1 | 2 | 7.41 | ['pri4', 'LNDTCs3'] | |  |
| Akap9 | 2 | 7.41 | ['LNDTCs2', 'LNDTCs6'] | |  |
| Apc | 2 | 7.41 | ['LNDTCs2', 'LNDTCs8'] | |  |
| Arid1a | 2 | 7.41 | ['BMDTCs2', 'LNDTCs8'] | |  |
| Asxl1 | 2 | 7.41 | ['pri7', 'BMDTCs8'] | |  |
| Asxl2 | 2 | 7.41 | ['BMDTCs9', 'LNDTCs9'] | |  |
| Atm | 2 | 7.41 | ['pri2', 'BMDTCs6'] | |  |
| Bcl11a | 2 | 7.41 | ['BMDTCs9', 'LNDTCs9'] | |  |
| Bcl9l | 2 | 7.41 | ['BMDTCs9', 'LNDTCs9'] | |  |
| Bcr | 2 | 7.41 | ['BMDTCs9', 'LNDTCs9'] | |  |
| Birc6 | 2 | 7.41 | ['LNDTCs2', 'LNDTCs4'] | |  |
| Braf | 2 | 7.41 | ['LNDTCs1', 'BMDTCs3'] | |  |
| Card11 | 2 | 7.41 | ['BMDTCs9', 'LNDTCs9'] | | |
| Cebpa | 2 | 7.41 | ['BMDTCs9', 'LNDTCs9'] | | |
| Chd2 | 2 | 7.41 | ['BMDTCs9', 'LNDTCs9'] | | |
| Crtc1 | 2 | 7.41 | ['BMDTCs9', 'LNDTCs9'] | | |
| Crtc3 | 2 | 7.41 | ['LNDTCs4', 'BMDTCs6'] | | |
| Ctnnd2 | 2 | 7.41 | ['BMDTCs2', 'BMDTCs8'] | | |
| Dcc | 2 | 7.41 | ['pri2', 'pri7'] |  | |
| Epas1 | 2 | 7.41 | ['pri7', 'pri3'] |  | |
| Etv4 | 2 | 7.41 | ['BMDTCs9', 'LNDTCs9'] | | |
| Ewsr1 | 2 | 7.41 | ['pri2', 'LNDTCs3'] | | |
| Fam135b | 2 | 7.41 | ['LNDTCs2', 'LNDTCs4'] | | |
| Fancd2 | 2 | 7.41 | ['pri2', 'BMDTCs7'] | | |
| Fat3 | 2 | 7.41 | ['pri2', 'LNDTCs4'] | | |
| Fcgr2b | 2 | 7.41 | ['pri6', 'BMDTCs9'] | | |
| Fgfr1 | 2 | 7.41 | ['BMDTCs9', 'LNDTCs8'] | | |
| Flt4 | 2 | 7.41 | ['pri2', 'pri7'] |  | |
| Foxl2 | 2 | 7.41 | ['BMDTCs9', 'LNDTCs9'] | | |
| Gnas | 2 | 7.41 | ['BMDTCs8', 'LNDTCs8'] | | |
| Grin2a | 2 | 7.41 | ['BMDTCs8', 'LNDTCs8'] | | |
| Hip1 | 2 | 7.41 | ['BMDTCs2', 'BMDTCs6'] | | |
| Hoxc11 | 2 | 7.41 | ['BMDTCs8', 'LNDTCs8'] | | |
| Ikzf1 | 2 | 7.41 | ['BMDTCs9', 'LNDTCs9'] | | |
| Kat6b | 2 | 7.41 | ['BMDTCs4', 'LNDTCs8'] | | |
| Kdm5c | 2 | 7.41 | ['BMDTCs7', 'BMDTCs6'] | | |
| Klf4 | 2 | 7.41 | ['BMDTCs9', 'LNDTCs9'] | | |
| Kmt2c | 2 | 7.41 | ['pri2', 'BMDTCs4'] | | |
| Lats2 | 2 | 7.41 | ['pri2', 'LNDTCs6'] | | |
| Lmna | 2 | 7.41 | ['BMDTCs9', 'LNDTCs9'] | | |
| Macc1 | 2 | 7.41 | ['BMDTCs8', 'LNDTCs8'] | | |
| Mafb | 2 | 7.41 | ['BMDTCs9', 'LNDTCs9'] | | |
| Mn1 | 2 | 7.41 | ['BMDTCs9', 'LNDTCs9'] | | |
| Nab2 | 2 | 7.41 | ['BMDTCs9', 'LNDTCs9'] | | |
| Nbea | 2 | 7.41 | ['pri2', 'BMDTCs3'] | | |
| Ncor1 | 2 | 7.41 | ['pri7', 'BMDTCs3'] | | |
| Numa1 | 2 | 7.41 | ['LNDTCs6', 'BMDTCs8'] | | |
| Polq | 2 | 7.41 | ['LNDTCs2', 'BMDTCs4'] | | |
| Prdm16 | 2 | 7.41 | ['BMDTCs8', 'LNDTCs8'] | | |
| Prrx1 | 2 | 7.41 | ['BMDTCs9', 'LNDTCs9'] | | |
| Ptch1 | 2 | 7.41 | ['pri2', 'pri4'] |  | |
| Ptprc | 2 | 7.41 | ['LNDTCs2', 'LNDTCs4'] | | |
| Rara | 2 | 7.41 | ['BMDTCs8', 'LNDTCs8'] | | |
| Ret | 2 | 7.41 | ['LNDTCs7', 'LNDTCs4'] | | |
| Rspo3 | 2 | 7.41 | ['BMDTCs9', 'LNDTCs9'] | | |
| Sall4 | 2 | 7.41 | ['pri7', 'LNDTCs8'] | | |
| Sbds | 2 | 7.41 | ['BMDTCs9', 'LNDTCs9'] | | |
| Sf3b1 | 2 | 7.41 | ['BMDTCs9', 'LNDTCs9'] | | |
| Sfrp4 | 2 | 7.41 | ['BMDTCs9', 'LNDTCs9'] | | |
| Sox2 | 2 | 7.41 | ['BMDTCs9', 'LNDTCs9'] | | |
| Ss18l1 | 2 | 7.41 | ['BMDTCs9', 'LNDTCs9'] | | |
| Stat5b | 2 | 7.41 | ['BMDTCs9', 'LNDTCs9'] | | |
| Tbx3 | 2 | 7.41 | ['BMDTCs9', 'LNDTCs9'] | | |
| Tmem127 | 2 | 7.41 | ['BMDTCs9', 'LNDTCs9'] | | |
| Trim27 | 2 | 7.41 | ['BMDTCs8', 'LNDTCs8'] | | |
| Vav1 | 2 | 7.41 | ['BMDTCs4', 'pri6'] | | |
| Wif1 | 2 | 7.41 | ['pri2', 'BMDTCs7'] | | |
| Wwtr1 | 2 | 7.41 | ['LNDTCs4', 'BMDTCs8'] | | |
| Zfhx3 | 2 | 7.41 | ['BMDTCs9', 'LNDTCs9'] | | |
