# Supplementary material for: Expression signature and molecular basis of CDH11 in OSCC detected by a combination of multiple methods
Source: BMC Med Genomics. 2023 Apr 3;16:70. doi: 10.1186/s12920-023-01499-7 (PMC10069064; doi:10.1186/s12920-023-01499-7)
Supplement: Supplementary file 3 — Supplementary Material 3 [file 12920_2023_1499_MOESM3_ESM.pdf]

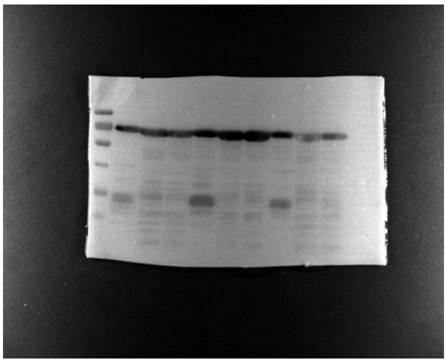

Figure S1:Full-length blots/gels from the western blot assay of CDH11.

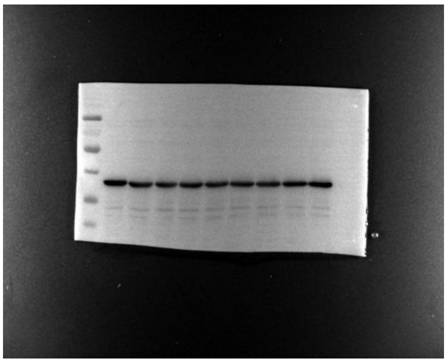

Figure S2:Full-length blots/gels from the western blot assay of GAPDH.

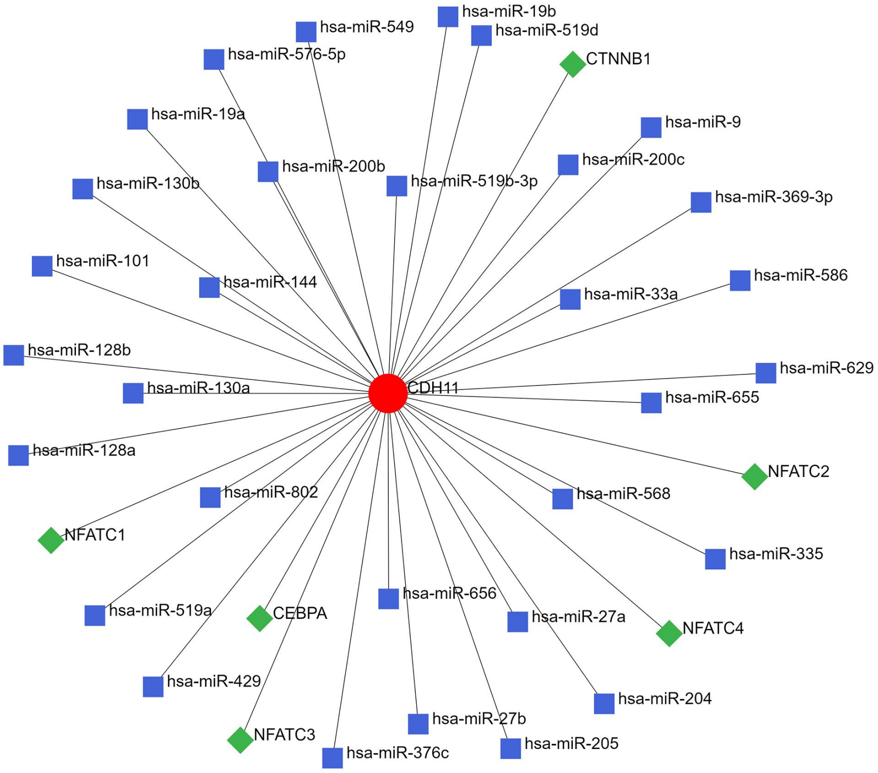

Figure S3: Transcription factor/miRNA-mRNA network of CDH11. The red circle patterns represent CDH11; blue square patterns represent up-stream miRNAs of CDH11; green diamond patterns represents up-stream transcriptional factors of CDH11;

A

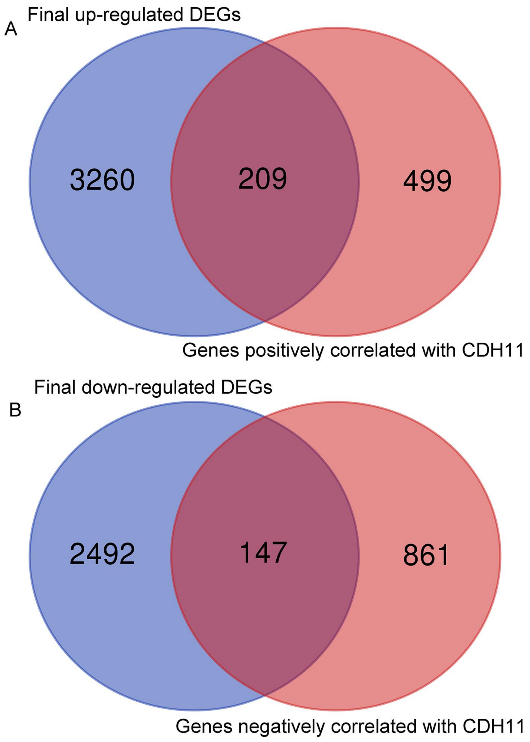

B

Figure S4: Co-expressed genes of CDH11. (A) Venn diagrams of up-regulated differentially expressed genes in oral squamous cell carcinoma and genes positively correlated with CDH11; blue represents 3,469 up-regulated differentially expressed genes, and red represents 209 genes positively correlated with CDH11. (B) Venn diagrams of down-regulated differentially expressed genes in oral squamous cell carcinoma and genes negatively correlated with CDH11. Blue represents 2,492 down-regulated differentially expressed genes and red represents 861 genes negatively correlated with CDH11. DEGs, differentially expressed genes;

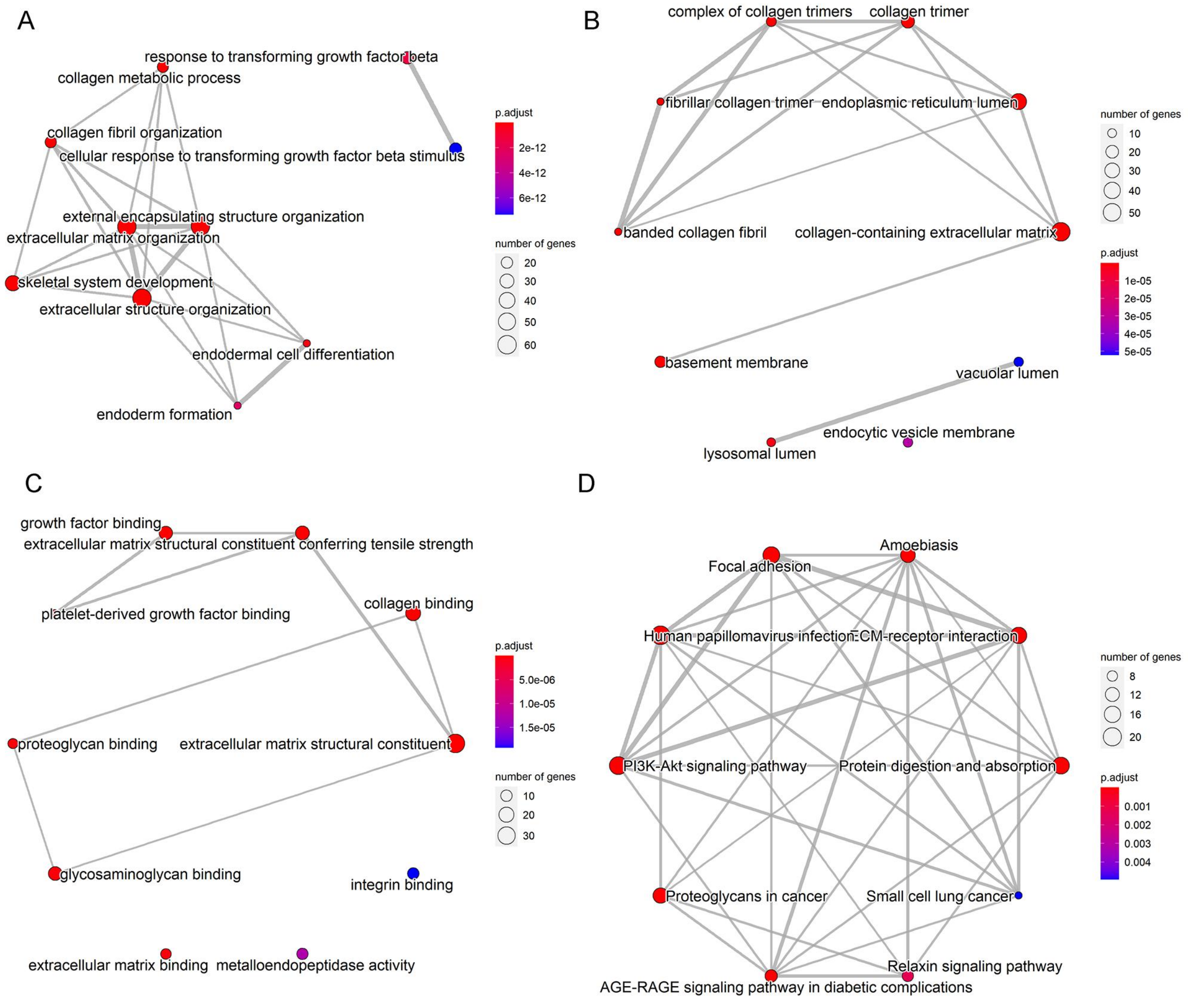

Figure S5: Gene ontology (GO) function annotation and Kyoto Encyclopedia of Genes and Genomes (KEGG) pathway analysis of genes positively co-expressed with CDH11. (A) Biological process emap plot of 209 genes positively co-expressed with CDH11. (B) Cell composition emap plot of 209 genes positively co-expressed with CDH11. (C) Molecular function emap plot of 209 genes positively co-expressed with CDH11. (D) KEGG emap plot of 209 genes positively co-expressed with CDH11;

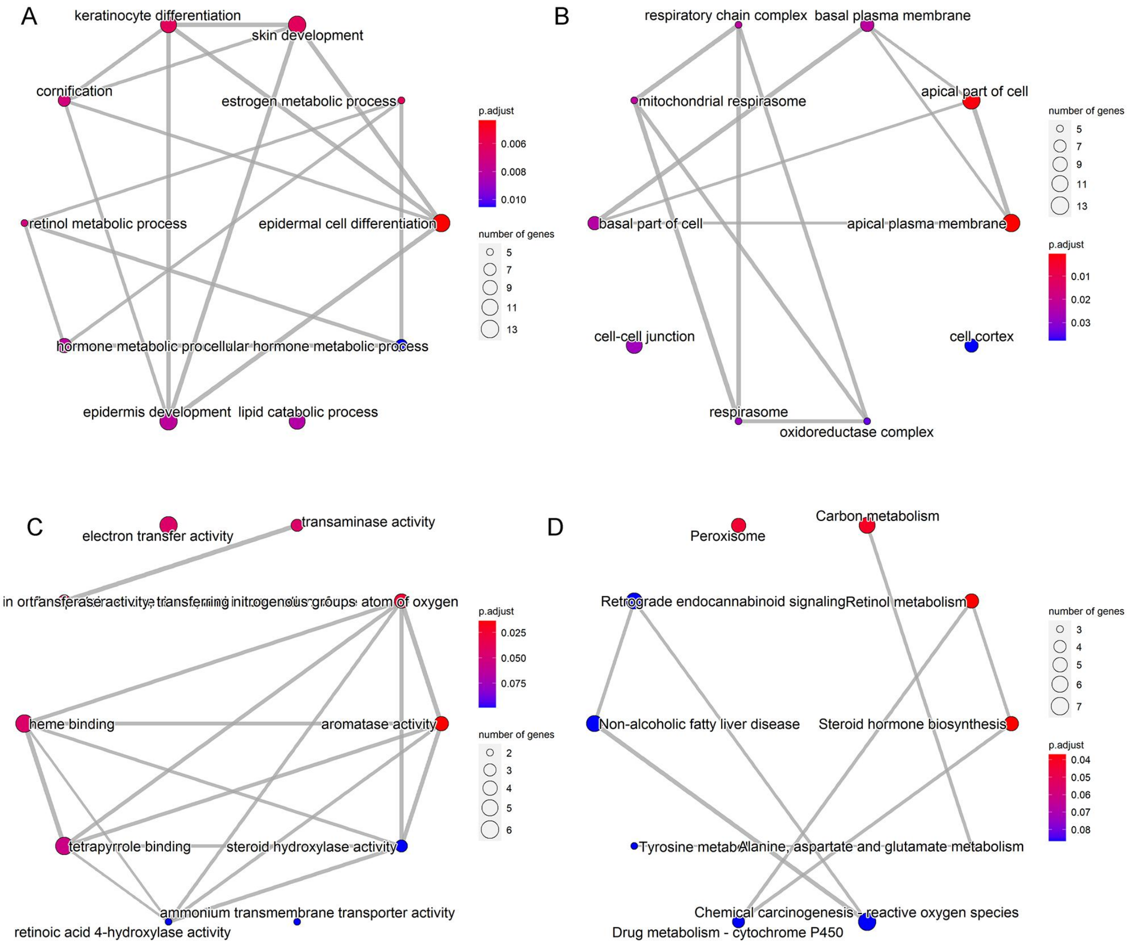

Figure S6: Gene ontology (GO) function annotation and Kyoto Encyclopedia of Genes and Genomes (KEGG) pathway analysis of genes negatively co-expressed with CDH11. (A) Biological process emap plot of 147 genes negatively co-expressed with CDH11. (B) Cell composition emap plot of 147 genes negatively co-expressed with CDH11. (C) Molecular function emap plot of 147 genes negatively co-expressed with CDH11. (D) KEGG emap plot of 147 genes negatively co-expressed with CDH11;
